# Biosynthesis of DHGA_12_ and its roles in *Arabidopsis* seedling establishment

**DOI:** 10.1038/s41467-019-09467-5

**Published:** 2019-04-16

**Authors:** Hao Liu, Siyi Guo, Minghua Lu, Yu Zhang, Junhua Li, Wei Wang, Pengtao Wang, Junli Zhang, Zhubing Hu, Liangliang Li, Lingyu Si, Jie Zhang, Qi Qi, Xiangning Jiang, José Ramón Botella, Hua Wang, Chun-Peng Song

**Affiliations:** 1 Key Laboratory of Plant Stress Biology, School of Life Sciences, Henan University, 475004 Kaifeng, China; 2 State Key Laboratory of Cotton Biology, School of Life Sciences, Henan University, 475004 Kaifeng, China; 3 College of Chemistry and Chemical Engineering, Henan University, 475004 Kaifeng, China; 4 College of Life Sciences, Henan Normal University, 453007 Xinxiang, China; 5 College of Biological Sciences and Biotechnology, Beijing Forestry University, 100083 Beijing, China; 6 Plant Genetic Engineering Laboratory, School of Agriculture and Food Sciences, The University of Queensland, Brisbane, QLD 4072 Australia; 7 Engineering Research Center for Nanomaterials, Henan University, 475004 Kaifeng, China

**Keywords:** Plant sciences, Plant hormones, Gibberellins

## Abstract

Seed germination and photoautotrophic establishment are controlled by the antagonistic activity of the phytohormones gibberellins (GAs) and abscisic acid (ABA). Here we show that *Arabidopsis thaliana* GAS2 (Gain of Function in ABA-modulated Seed Germination 2), a protein belonging to the Fe-dependent 2-oxoglutarate dioxygenase superfamily, catalyzes the stereospecific hydration of GA_12_ to produce GA_12_ 16, 17-dihydro-16α-ol (DHGA_12_). We show that DHGA_12_, a C_20_-GA has an atypical structure compared to known active GAs but can bind to the GA receptor (GID1c). DHGA_12_ can promote seed germination, hypocotyl elongation and cotyledon greening. Silencing and over-expression of *GAS2* alters the ABA/GA ratio and sensitivity to ABA during seed germination and photoautotrophic establishment. Hence, we propose that GAS2 acts to modulate hormonal balance during early seedling development.

## Abstract (web summary)

Gibberellins are a major class of phytohormones that regulate plant growth and development. Here the authors show that the *Arabidopsis* GAS2 protein catalyses production of DHGA_12_, an atypical bioactive GA, and show that GAS2 regulates ABA sensitivity during seed germination and early development.

## Introduction

Seed germination and subsequent photoautotrophic development of seedlings are complex early developmental processes crucial to subsequent success in the plant life cycle^1^. The antagonistic roles of the phytohormones abscisic acid (ABA) and gibberellins (GAs) in seed germination and seedling development, and their complex regulatory networks, have been the focus of intense research^2^. It is widely accepted that ABA promotes the onset of dormancy and inhibits germination, whereas GA prevents dormancy and stimulates germination^3–6^. The overall ABA/GA balance in the seed is tightly controlled, since it ultimately dictates its developmental fate, and this balance can be altered by modifying the ratio of endogenous ABA/GA levels, and/or affecting intrinsic sensitivities to either hormone^6^. After germination, seedlings must rapidly develop efficient root systems and establish photoautotrophic capability to adapt to their environment, thereby maximizing their chances of survival^7^. When young seedlings emerge from the ground, they experience dramatic environmental changes that trigger multiple developmental processes, including cotyledon opening, chloroplast development, and leaf de-etiolation. The molecular mechanisms that control the ABA/GA balance in response to environmental factors during the heterotrophic-to-autotrophic transition are mostly unexplored^8^.

Existing GAs are derivatives of diterpenoid carboxylic acids, and possess a C3-hydroxyl group^9^. The early steps of biosynthesis, involving *ent*-copalyl diphosphate synthase (CPS), *ent*-kaurene synthase (KS) and *ent*-kaurene oxidase (KO), are each encoded by a single gene in *Arabidopsis*. Mutants defective in these genes (*ga1*, *ga2*, and *ga3*) display severe GA-deficient dwarfing^10^. In contrast to the early GA-biosynthetic enzymes, the GA 20-oxidases (GA20oxs) and GA 3-oxidases (GA3oxs) catalyzing the late steps of the pathway belong to separate gene families within the 2-oxoglutarate-dependent dioxygenases (2ODDs). Their *Arabidopsis* mutants (*ga4* and *ga5*) develop a semi-dwarf phenotype and can germinate without the addition of exogenous GAs^11^. Considerable progress has been made in the discovery of naturally occurring GA structures, with their numbers now reaching 136, although their physiological functions are mostly unknown^12^. Remarkably, no novel GAs or GA modification routes linked to developmental regulation have been discovered in over 10 years, although it is logical to hypothesize the existence of additional undiscovered natural GAs as well as novel routes to allow their precise regulation.

GA20ox proteins have been identified in a large variety of plants^13^. Of the five *Arabidopsis* GA20oxs, four (GA20ox1, GA20ox2, GA20ox3, and GA20ox4) have GA20ox activity, whereas GA20ox5 has only partial activity^14,15^. Expression profiles and mutant analysis indicated functional redundancy of *AtGA20ox1*, *AtGA20ox2*, and *AtGA20ox3*, with all playing key roles in GA biosynthesis and plant development. In contrast, the low expression levels of *AtGA20ox4* and *AtGA20ox5* explain their less prominent role in development^14^. Although the pathways for the synthesis of bioactive GAs catalyzed by the GA20ox subfamily have been extensively studied^16,17^, the roles of more than 100 remaining 2ODD gene family members are still largely unknown^18^.

Here, we describe the characterization of “Gain-of-function in ABA-modulated Seed germination 2” (*GAS2*, hereafter) that shows lowered sensitivity to ABA in germination and early seedling development in overexpressing lines than WT, but enhanced ABA sensitivity in loss-of-function mutants of *GAS2*. It encodes a Fe-dependent 2-oxoglutarate dioxygenase that catalyzes the biosynthesis of an atypical bioactive GA, named GA_12_ 16, 17-dihydro-16α-ol (DHGA_12_). We show that DHGA_12_ can bind the GA receptor and to a certain extent promotes seed germination and hypocotyl elongation, as well as enhancing cotyledon greening and seedling development. We propose that GAS2 modulates both the ABA/GA ratio and ABA sensitivity influencing early developmental events in seedlings. Further characterization suggests the possibility that DHGA_12_ is involved in the modulation of seedling establishment.

## Results

### *GAS2* negatively regulates ABA sensitivity

**Fig. 1:**
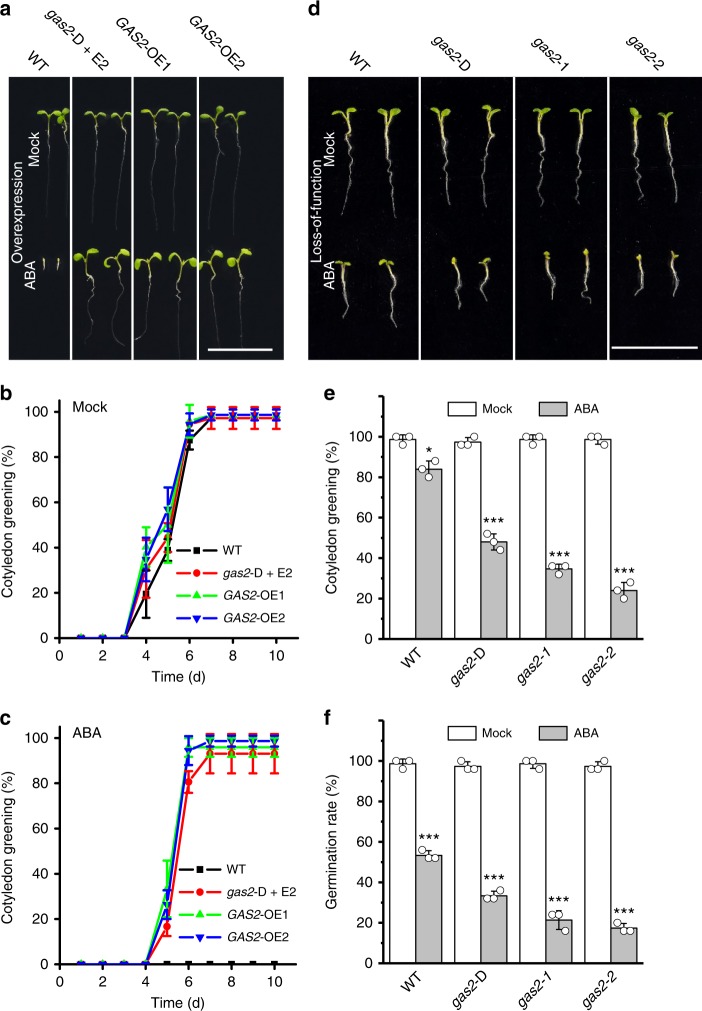
*GAS2* modulates the sensitivity to ABA in seed germination and seedling establishment. **a** Phenotypes of 10-d-old seedlings of wild-type, E2-induced *gas2*-D and two *GAS2* overexpression transgenic lines (*GAS2*-OE1 and OE2), grown on MS in absence (Mock) or presence of 0.5 µM ABA (ABA). Bar = 1.5 cm. **b**, **c** Cotyledon greening analysis of 10-d-old seedlings of wild-type, E2-induced *gas2*-D and two *GAS2* overexpression lines (*GAS2*-OE1 and OE2) grown on MS with or without the addition of 0.5 µM ABA. Error bars represent SD (standard deviations) (*n* = 72). **d** Phenotypes of 8-d-old seedlings of wild-type, noninduced *gas2*-D, *gas2-1* and *gas2-2*, grown on MS in absence (Mock) or presence of 0.2 µM ABA (ABA). Bar = 1.5 cm. **e** Cotyledon greening analysis of 8-d-old seedlings of wild-type, noninduced *gas2*-D, *gas2-1* and *gas2-2* grown on MS in absence (Mock) or presence of 0.2 µM ABA (ABA). Error bars represent SD (standard deviations) (*n* = 72). **f** Seed germination analysis of wild-type, noninduced *gas2*-D, *gas2-1* and *gas2-2*, grown on MS in absence (Mock) or presence of 0.2 µM ABA (ABA) at 48 h. Error bars represent SD (standard deviations) (*n* = 72). E2 stands for 17-β-estradiol. *P < 0.05, ***P < 0.001, *t* test versus mock (**e** and **f**). Source data are provided as a Source Data file. ABA abscisic acid, MS Murashige and Skoog agar

With the aim of finding modulators of ABA signaling, we produced an extensive collection of transgenic *Arabidopsis* lines carrying the chemically (17-β-estradiol, E2) inducible XVE system^19^ adjacent to the T-DNA border. Preliminary experiments established 0.5 µM ABA as the optimal concentration for screening based on its inhibition of seed germination and cotyledon greening (Supplementary Fig. 1a). Screening of 38,000 T-DNA seeds sown onto Murashige and Skoog (MS) agar plates supplemented with 0.5 µM ABA and 5 µM E2 identified 35 ABA-insensitive plants. One of these showed strong ABA insensitivity during germination and seedling development, and was named Gain of Function in ABA-modulated Seed Germination 2 (*gas2*-D) (Fig. 1a, c). Homozygous seeds were obtained for the *gas2*-D T-DNA line and re-tested under more stringent conditions (1 µM ABA) to confirm the E2-inducible phenotype (Supplementary Fig. 1b). Seed germination and cotyledon development was strongly inhibited in wild-type (WT) and noninduced *gas2*-D plants plated on MS medium supplemented with either 0.2, 0.5 or 1 µM ABA, whereas the addition of E2 blocked the effect of ABA (Fig. 1a–f and Supplementary Figs. 1b, 2a–d). Growth of wild-type seedlings was arrested by 0.5 µM ABA even when treatment was done following radicle emergence (Fig. 1a, c). In contrast, treatment with ABA did not significantly delay germination of *gas2*-D seeds, nor did it arrest the autotrophic transition and seedling establishment of *gas2*-D under E2-inducing conditions (Fig. 1a, c). No obvious differences in germination and early seedling development were apparent between E2-induced *gas2*-D and WT plants grown on MS medium (Fig. 1a, b).

The position of the T-DNA insertion in the *gas2*-D line was determined by thermal asymmetric interlaced polymerase chain reaction (TAIL-PCR), establishing its location in the promoter region of the *Arabidopsis* gene At2g36690*/GAS2* (Supplementary Fig. 1c–e) with the trans-activator region localized ~157 bp upstream of the *GAS2* start codon (Supplementary Fig. 1c). *GAS2* encodes a member of the 2ODD protein family, showing 45.6% sequence identity to GA20ox1 in the conserved DIOX_N (Non-haem dioxygenase N-terminal domain) and 2OG-FeII_Oxy (Oxoglutarate/iron-dependent dioxygenase) domain (Supplementary Fig. 1g)^16,17^. Expression analysis confirmed that *GAS2* transcript levels in *gas2-*D plants were highly induced by treatment with E2, being 5-6 folds higher than WT (Supplementary Fig. 1f). Interestingly, the proximity of the T-DNA to the start of transcription resulted in almost complete silencing of *GAS2* in the absence of the inducer E2 (Supplementary Fig. 1f).

To confirm that the ABA-insensitive phenotype was due to the overexpression of *GAS2*, ten independent transgenic lines were produced by placing the *GAS2* cDNA under the control of the cauliflower mosaic virus (CaMV) *35S* promoter and two independent lines, *GAS2*-OE1 and *GAS2*-OE2, with high *GAS2* expression levels (Supplementary Fig. 1f) were selected for further study. The *GAS2*-OE lines displayed an ABA-insensitive phenotype similar to that observed for the gain-of-function *gas2*-D line in the presence of E2 (Fig. 1a–c).

The noninduced *gas2*-D line was treated as a knockdown mutant given the strong downregulation of *GAS2* expression caused by the T-DNA insertion (Supplementary Fig. 1c, f). We produced two more loss-of-function mutants using CRISPR/Cas9 technology (*gas2-1* and *gas2-2*, hereafter). Sequencing of T2 homozygous lines identified a 1 bp deletion in *gas2-1* and a 1 bp insertion in *gas2-2*, leading in each case to frameshifts and complete *GAS2* loss-of-function (Supplementary Fig. 1c). ABA-induced inhibition of cotyledon development and seed germination was more severe in the knockdown (*gas2*-D) plants than WT. As expected, these ABA-induced phenotypes were even more pronounced in the *gas2-1* and *gas2-2* lines than *gas2*-D (Fig. 1d–f and Supplementary Fig. 2a–d). Overall, our data show that loss-of-function or knockdown mutants of *GAS2* are slightly hypersensitive to ABA during germination and early seedling development, whereas the gain-of-function and overexpressing lines are less sensitive to ABA than WT (Fig. 1 and Supplementary Fig. 2), suggesting that GAS2 negatively regulates ABA sensitivity during germination, phototrophic establishment and seedling development.

### GAS2 functions in germination and early seedling development

**Fig. 2:**
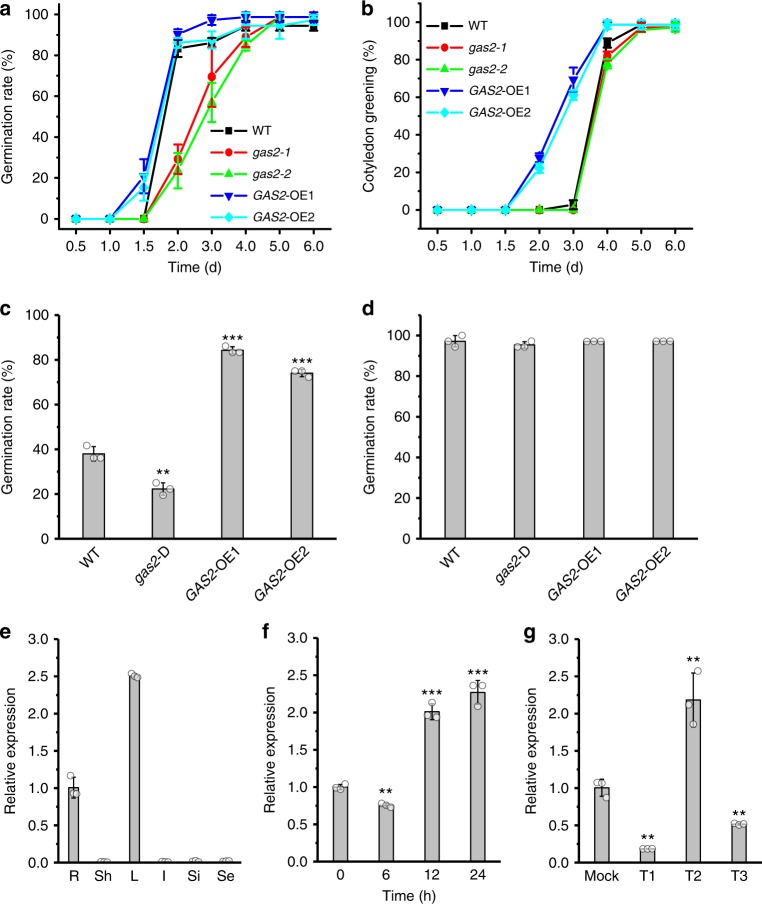
Silencing and overexpression of *GAS2* affects seed germination and cotyledon greening. **a**, **b** Seed germination and cotyledon greening analysis of wild-type, *gas2-1*, *gas2-2* and two *GAS2* overexpression lines (*GAS2*-OE1 and OE2) grown on MS. Error bars represent SD (standard deviations) (*n* = 72). **c**, **d** Germination analysis of wild-type, noninduced *gas2*-D and overexpressing *GAS2* fresh seeds (not allowed to dry) at the 2-d timepoint after sowing with no stratification (**c**) and stratification (at the 3-d timepoint after sowing) (**d**). Error bars represent SD (*n* = 108). **p *<* 0.01, ***p *<* 0.001, *t* test. **e** Relative *GAS2* mRNA levels in roots (R), shoots (Sh), leaves (L), inflorescences (I), siliques (Si) and seeds (Se) analyzed by RT-qPCR. Error bars represent SD. **f** Relative *GAS2* mRNA levels in hypocotyls of *Arabidopsis* after different light/dark treatment (dark 9 d, dark 9 d + light 6 h, dark 9 d + light 12 h and dark 9 d + light 24 h). The dark 9-d level was arbitrarily adjusted to 1 and the remaining levels were normalized to that value. Error bars represent SD. **p < 0.01, ***p < 0.001, *t* test. **g** Relative *GAS2* mRNA levels of the seedlings in response to red and far-red light exposure, analyzed by RT-qPCR. Error bars represent SD. **p < 0.01, *t* test. Mock: Far red light 0 h; T1: Far red light 2 h; T2: Far red light 2 h + red light 2 h; T3: Far red light 2 h + red light 2 h + Far red light 2 h. Source data are provided as a Source Data file. MS Murashige and Skoog agar

Phenotypic analysis of transgenic *Arabidopsis* *GAS2* knockout and overexpressing lines showed clear alterations in germination and early seedling development. When seeds were allowed to fully dry for several weeks after harvesting, germination of *gas2-1* and *gas2-2* seeds was slower than WT seeds (29 and 23% vs. 83%, 48 h after stratification in MS) (Fig. 2a). In contrast, *GAS2*-OE1 and *GAS2*-OE2 overexpression lines showed faster germination than WT (21 and 15% vs. 0% under the same conditions 36 h after stratification in MS) (Fig. 2a).

Similar differences were also evident when fresh seeds (not allowed to dry) were analyzed (Fig. 2c). In the absence of stratification, *gas2*-D seeds, with strong *GAS2* downregulation, showed a 50% reduction in germination compared with WT 48 h after sowing, whereas the germination rates of *GAS2*-OE1 and *GAS2*-OE2 lines were twice that of the WT (Fig. 2c). All lines achieved 100% germination 3 days after stratification (Fig. 2d). Early seedling development, measured as the percentage of cotyledon greening, was also altered in the transgenic lines with *GAS2*-OE1 and *GAS2*-OE2 overexpressing lines showing 69 and 62% values 3 days after germination, as compared with 3% for WT seedlings; the mutant *gas2-1* and *gas2-2* lines showed a delay in cotyledon greening (Fig. 2b).

As mentioned above, GA 20-oxidases belong to a large class of Fe-containing enzymes, found in plants and fungi, that share a common mechanism of action^18^. Bioinformatics analysis revealed that GAS2 is a member of the 2OG-Fe-dependent oxygenase family, distantly related to GA20oxs (Supplementary Fig. 3). Five GA20ox enzymes have been characterized in *Arabidopsis*, and three of them play important roles in the regulation of active GA levels, having profound effects on vegetative development^14^. Phylogenetic analysis reveals that GAS2 belongs to a subfamily (designated as the GAS2 subfamily) different to that of the GA 20-oxidases.

To assess *GAS2* expression, we performed reverse transcription quantitative polymerase chain reaction (RT-qPCR) analysis using samples taken from different* Arabidopsis* tissues. Very low levels of *GAS2* expression were detected in all tissues, with the exception of roots and leaves (Fig. 2e). These results are consistent with *Arabidopsis* microarray data archived in the BAR eFP browser (http://bar.utoronto.ca/efp/cgi-bin/efpWeb.cgi) (Supplementary Fig. 4a, b)^20^. Previous microarray expression studies indicate that light induces the expression of *GAS2* under long day conditions (Supplementary Fig. 4c). Plants kept in darkness for 9 days displayed an initial reduction in *GAS2* transcript levels 6 h after light exposure, followed by a dramatic increase after 12 h of continuous light with high levels maintained after 24 h (Fig. 2f). It is well established that *Arabidopsis* germination is inhibited by far-red light and promoted by red light^21,22^ consistent with our results showing that *GAS2* expression was repressed by far-red light whereas treatment with red light reversed the effect, inducing expression two folds over normal levels (Fig. 2g). Characterization of transgenic *Arabidopsis* lines carrying 3.96 kb of the *GAS2* genomic region, including the promoter and gene sequences fused to the β-glucuronidase (GUS) reporter gene (Supplementary Fig. 5a–c) showed induction of GUS staining by ABA whereas GA_4_ treatment almost completely abolished it (Supplementary Fig. 5a–c).

### GAS2 catalyzes the hydration of GA_12_

**Fig. 3:**
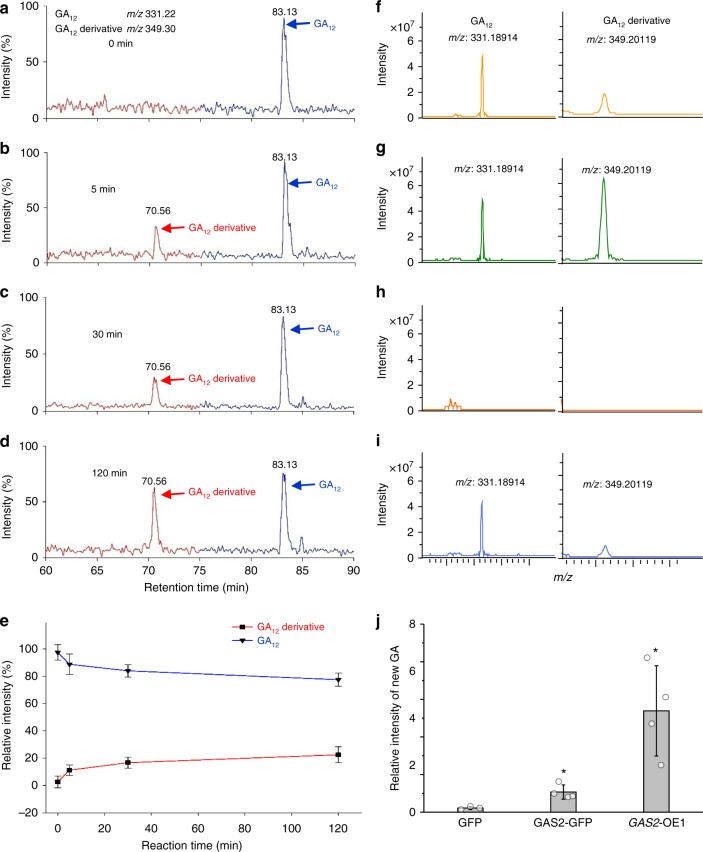
Analysis of products produced by the catalytic conversion of GA_12_ by GAS2 in vivo and in vitro. **a**–**d** LC-MS base peak chromatogram of the products generated by the catalytic conversion of GA_12_ by GAS2 at different time intervals (0, 5, 30, and 120 min). [17,17-^2^H_2_]-GA_12_ was used in this assay. **e** LC-MS dynamic analysis of GA_12_ and GA_12_ derivative in the reaction solution at different time intervals (0, 5, 30, and 120 min). LC-MS liquid chromatography/mass spectrometry. **f**–**i** GA_12_ and GA_12_ derivative detection using MALDI FTICR-MS spectra in *35S::GAS2-GFP* (GAS2-GFP) (**f**), *35S::GFP* (**i**) transiently transformed in protoplasts and *GAS2-*OE1 plants (**g**) with GA_12_ (2.5 μg/mL), *35S::GFP* transiently transformed into protoplasts was used as a negative control without GA_12_ treatment (**h**). **j** Relative intensity of GA_12_ derivative in *Arabidopsis* protoplasts transiently transformed with *35S::GAS2-GFP* (GAS2-GFP) and 35S::GFP (GFP) overnight. The protoplasts from *GAS2*-OE1 plants (GAS2-OE1) were used as a control. GA_12_ (2.5 μg/mL) was supplemented as the substrate for GA_12_ derivative synthesis catalyzed by GAS2. Values shown are means ± SD (*n* = 3). *p < 0.05, *t* test. Source data are provided as a Source Data file

To investigate whether GAS2 exhibits enzymatic activity in GA biosynthesis, we designed several in vitro and in vivo experiments. In a first set of experiments, we performed in vitro biosynthetic assays using the purified GAS2 protein, cofactors, and three different deuterium-labeled substrates. Reaction mixtures containing GA_12_ as substrate were collected 0, 5, 30 and 120 min after the start of the reaction. Liquid chromatography-mass spectrometry (LC-MS) analysis showed the gradual appearance of a newly formed peak with a retention time of 70.56 min (Fig. 3a–d), and a progressive decline in GA_12_ levels (Fig. 3e). Negative control reactions using either denatured GAS2, lacking cofactors, or containing EDTA as an Fe chelator failed to yield any products (Supplementary Fig. 6a–e). No catalytic products were detected in the reactions containing GA_15_ or GA_24_ as substrates when analyzed by LC-MS (Supplementary Fig. 7a–h). To investigate whether GA_12_ can act as an endogenous substrate for GAS2, we transiently expressed a GAS2-GFP fusion (*35S::GAS2-GFP*) in WT *Arabidopsis* protoplasts in medium containing GA_12_. MALDI FTICR-MS analysis revealed the appearance of a peak with of *m/z* 349.201, obtained from full-scan MS (Fig. 3f), identical to the synthetic GA_12_ derivative standard (Supplementary Fig. 8). Transient expression of *35S::GFP* in the presence of GA_12_ produced a small amount of the GA_12_ derivative, perhaps due to enzymatic conversion of GA_12_ by endogenous GAS2 (Fig. 3i). When protoplasts from the *GAS2*-OE1 overexpressing line were incubated with GA_12_, a very strong GA_12_ derivative peak was observed (Fig. 3g) consistent with the high expression levels observed in this line (Supplementary Fig. 1f). As a negative control, transient expression of *35S::GFP* in WT protoplasts in the absence of GA_12_ did not produce any detectable GA_12_ derivative. Quantification of the relative GA_12_ derivative peak intensities shows the highest values for the *GAS2*-OE1 lines. This is as expected, since strong *GAS2* expression is seen in all protoplasts, and the lower values observed in the transient expression experiments using *35S::GAS2-GFP* can be explained by the relatively low efficiency of protoplast transformation (Fig. 3g). The control experiment, transient expression of GFP in the presence of GA_12_ produced very low levels of GA_12_ derivative (Fig. 3g).

To further investigate whether GA_12_ is an endogenous substrate for GAS2, we transiently expressed a GAS2-GFP fusion protein (*35S::GAS2-GFP*) in tobacco (*Nicotiana tabacum*) leaves. Protoplasts isolated from these leaves were incubated in medium containing GA_12_. LC-MS analysis identified appearance of a peak of an *m/z* 349.20 at a retention time of 2.04 min (Supplementary Fig. 9). In contrast, transient expression of *35S::GFP* in tobacco leaves in the presence of GA_12_ did not produce any peaks of *m/z* 349.20 at the 2.04 min retention time (Supplementary Fig. 9). Taken together, MALDI FTICR-MS and LC-MS data demonstrate that the presence of the GA_12_ derivative is caused by enzymatic conversion of GA_12_ by endogenous GAS2 (Fig. 3 and Supplementary Fig. 8). In addition, we detected natural occurrence of this GA_12_ derivative in maize seedlings proving that GA_12_ derivative is present in other plant species (Supplementary Fig. 10). Together, our results demonstrate that GAS2 can use GA_12_ as substrate for production of an intermediate in GA biosynthesis both in vivo and in vitro.

### Identification of DHGA_12_ structure

**Fig. 4:**
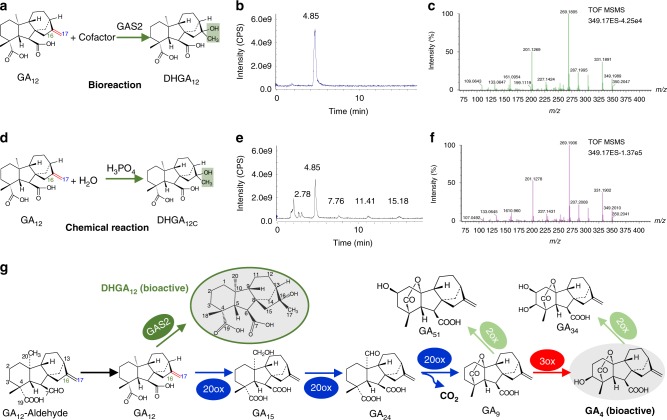
LC-MS analysis of the new gibberellin, DHGA_12_ and its position within the GA biosynthetic pathway. **a** Schematic of the GAS2-catalyzed conversion of GA_12_ to DHGA_12_. Cofactor: 24 µL, containing 133 mM 2-oxoglutarate, 133 mM ascorbate, 16.7 mM FeSO_4_, and 33.3 mg/mL catalase. **b** Total ion chromatogram (TIC) of DHGA_12_ by LC-MS. **c** MS spectrum of the peak at 4.85 min (retention time) in (**b**). **d** Schematic of the chemical synthesis of DHGA_12C_ (subscript C denotes chemically synthesized DHGA_12_). The chemical synthesis was conducted in the phosphoric acid solution (0.125 mol/L) at 60 ^o^C for 8 h. **e** TIC of DHGA_12C_ by LC-MS. **f** MS spectrum of the peak at 4.85 min (retention time) in (**e**). **g** The stereochemical configuration of DHGA_12_ (green oval) and gibberellin biosynthetic pathway including the GAS2-catalyzed production of DHGA_12_. 20ox GA 20-oxidase, 3ox GA 3-oxidase, 2ox GA 2-oxidase, LC-MS liquid chromatography/mass spectrometry, GA gibberellins, MS Murashige and Skoog agar

Purified products from the biosynthetic reactions were analyzed by LC-MS showing a characteristic retention time of 4.85 min (Fig. 4b). The molecular formula of the GA_12_ derivative (GA_12_ 16,17-dihydro-16α-ol, DHGA_12_) was inferred to be C_20_H_30_O_5_ from the addition of H_2_O to GA_12_ resulting in the hydration of the 16, 17-double bond (Fig. 4a–c and Supplementary Fig. 11). The existence of GA_12_ hydration activity for GAS2 was surprising, and we therefore sought further confirmation of our findings. Analysis of the product from a separate chemical synthesis reaction supported our findings (Fig. 4d–f). The major product structure of the synthesis reaction according to Markovnikov’s rule should be identical to DHGA_12_ (hereafter named DHGA_12C_, with the addition of the C subscript to denote chemically synthesized DHGA_12_) (Fig. 4a, d), which has been widely reported in previous studies as a classical electrophilic addition reaction^23,24^. DHGA_12_ and DHGA_12C_ have identical retention times (Fig. 4b, e) and TOF MS/MS spectra (Fig. 4c, f), consistent with identical molecular structures. For further confirmation, we performed NMR assays with the product from chemical synthesis, including ^1^H NMR,^13^C NMR,^1^H-^1^H NOESY,^1^H-^1^H COSY,^1^H-^13^C HMQC, and ^1^H-^13^ C HMBC spectra. From the corresponding 2D NOESY spectrum of DHGA_12_, cross-peaks between the proton H of C-14/C-15 and the proton H of C-17 are not observed, indicating a hydroxyl group on C-16α (Supplementary Figs. 12–19). DHGA_12_ is a C_20_-GA lacking the 4,10-lactone and a hydroxyl group (-OH) at C-3 in the β-orientation characteristic of traditional bioactive GAs (e.g. GA_1_, GA_3_, GA_4_ and GA_7_) (Fig. 4g inset a green oval). Searches in available databases and literature failed to find a compound with a structure and stereochemical configuration identical to DHGA_12_, therefore identifying it as an atypical gibberellin^12,25,26^.

In vivo subcellular localization experiments indicate that GAS2 is located in the cytoplasm (Supplementary Fig. 20), which is consistent with it acting on a C_20_-GA intermediate^27,28^. Our data demonstrate that GAS2 exhibits hydration activity using GA_12_ as a substrate to produce DHGA_12_. The proposed pathway for DHGA_12_ biosynthesis is illustrated in Fig. 4g. In terms of the general GA biosynthetic and catabolic pathway, DHGA_12_ is synthesized from GA_12_ in a reaction catalyzed by GAS2 in the cytoplasm, and represents a new branch in the pathway (Fig. 4g).

### DHGA_12_ is a bioactive GA

**Fig. 5:**
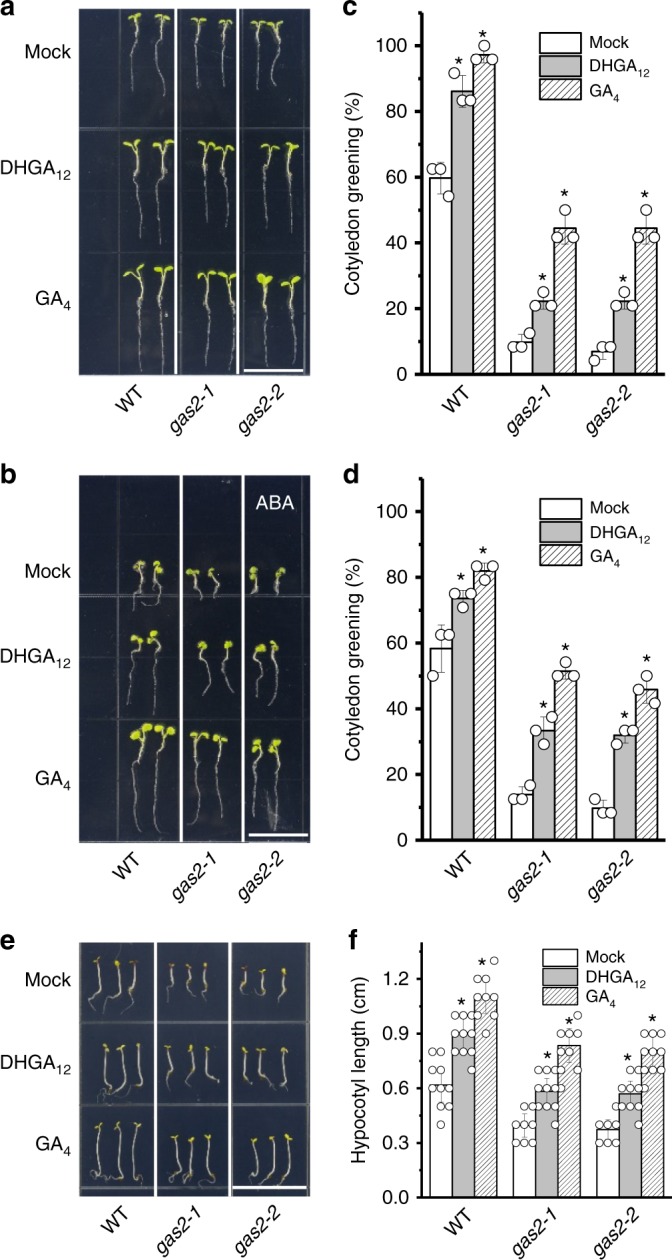
DHGA_12_ antagonistically suppresses the effect of ABA in seedling establishment. **a**, **b** Effects of exogenous application of DHGA_12_ and GA_4_ on cotyledon greening of wild-type, *gas2-1*, and *gas2-2*, germinated 10-d on MS and MS + 0.2 µM ABA, with or without the addition of DHGA_12_ or GA_4_. Bars = 1.5 cm. **c**, **d** Cotyledon greening analysis of wild-type, *gas2-1*, and *gas2-1*, grown on MS at day 4 (**c**) and MS + 0.2 µM ABA at day 7 (**d**), with or without the addition of DHGA_12_ or GA_4_. Error bars represent SD (*n* = 72, *t* test, *P < 0.05). Values are the mean of three independent experiments. **e**, **f** Effects of exogenous application of DHGA_12_ and GA_4_ on hypocotyl elongation of 5-d wild-type, *gas2-1* and *gas2-2* seedlings grown under continuous far-red light illumination. Data represent means ± SD (*n* = 22, *t* test, *P < 0.05). Source data are provided as a Source Data file. ABA abscisic acid

Since GAS2 can catalyze formation of DHGA_12_ from GA_12_ (Fig. 4g), and given the observed phenotypes for *gas2* and *GAS2-*OE, we postulated that DHGA_12_ is a bioactive GA, and alterations in DHGA_12_ could affect seedling development and ABA responses. To test our hypothesis, we first investigated the effect of exogenous application of DHGA_12_ on *Arabidopsis* cotyledon greening, using seedlings grown in MS medium supplemented with 0 and 0.2 µM ABA (Fig. 5a–d and Supplementary Fig. 21). As seen in *GAS2*-OE plants, exogenous application of DHGA_12_ to WT seedlings stimulates cotyledon greening and counteracts the inhibitory effect of ABA. Furthermore, the delayed cotyledon greening observed in *gas2-1* and *gas2-2* mutants was partially rescued by the addition of exogenous DHGA_12_. Similar effects were also observed following exogenous application of GA_4_, a GA known to be bioactive (Fig. 5a–d and Supplementary Fig. 21). To test whether DHGA_12_ is bioactive in hypocotyl elongation, seeds of wild-type, and *gas2-1* and *gas2-2* mutants, were directly plated on MS, with or without the addition of GA_4_ or DHGA_12_, and grown under continuous far-red light (FRL) illumination. Treatment with GA_4_ or DHGA_12_ significantly promoted hypocotyl elongation in all the tested genotypes (Fig. 5e, f). Promotion of elongation was less effective in *gas2-1* and *gas2-2* mutants than in WT plants. In addition, the effects of DHGA_12_ on plant growth and ABA response were less pronounced than that of GA_4_.

**Fig. 6:**
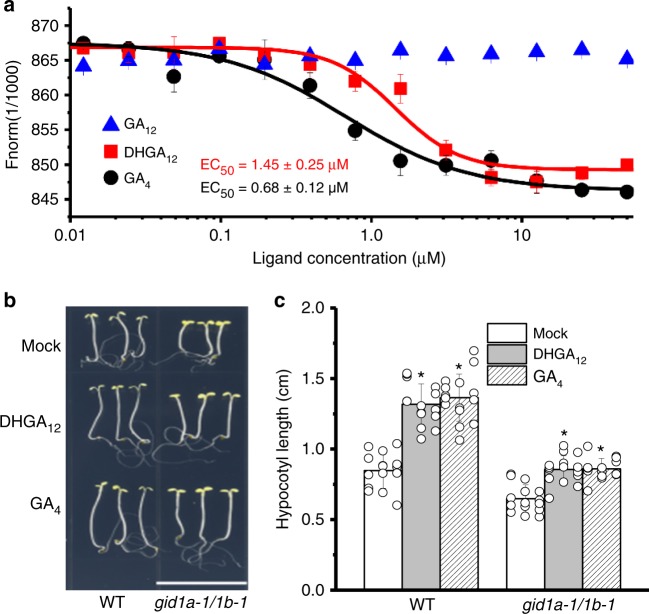
DHGA_12_ can directly bind to the GA receptor (GID1c). **a** Microscale thermophoresis (MST) analysis of DHGA_12_ and GA4 binding to GID1c. Dissociation constants of DHGA_12_ and GA_4_ with GID1c are 1.45 ± 0.25 μM and 0.68 ± 0.12 μM, respectively. Error bars represent SD (*n* = 3). **b**, **c** Effect of exogenous application of 5 μM DHGA_12_ and 5 μM GA_4_ on hypocotyl elongation of 10-d wild-type and *gid1a-1/1b-1* seedlings grown under continuous far-red light illumination. Data represent means ± SD (*n* = 15; *t* test; *P < 0.05). Bar = 1.5 cm. Source data are provided as a Source Data file. GA gibberellins

GA_4_, as a bioactive GA, has been shown to bind the GA receptor (GID1) to perform its biological roles^29^. Since DHGA_12_ may play a role as a bioactive GA in seedling development, we tested whether it can bind GID1. Firstly, we analyzed the structural similarities between DHGA_12_ and two bioactive GAs (GA_3_ and GA_4_) and its precursor GA_12_. Although comparison of the chemical structure of DHGA_12_ with those of other bioactive GAs (GA_3_ and GA_4_) demonstrated a different structure (Supplementary Fig. 22a–d), the DHGA_12_ molecule still shared considerable structural similarities. Further in silico analysis of the GID1a-DHGA_12_ complex suggested a binding energy of −8.39 kcal/mol, indicating thermodynamic conditions favorable for binding between DHGA_12_ and the GA receptor (Supplementary Fig. 22e). This was confirmed by a direct DHGA_12_ to GID1 binding assays using microscale thermophoresis (MST). A functional GID1 homolog, GID1c was purified and tested for its binding to DHGA_12_, GA_4_, and GA_12_ (a non-bioactive GA that does not bind the receptor). Our results showed that the glutathione S-transferase (GST)-tagged GID1c binds DHGA_12_ and GA_4_ with dissociation constants EC_50_ = 1.45 ± 0.25 µM and EC_50_ = 0.68 ± 0.12 µM (± indicates standard deviation, *n* = 3), respectively, whereas no binding of GA_12_ was detected (Fig. 6a).

To further investigate the biological relationship between DHGA_12_ and GID1, we employed a double GA receptor mutant (*gid1a-1/gid1b-1*) for genetic analyses. Treatment with exogenous DHGA_12_ and GA_4_ significantly promoted hypocotyl elongation of wild-type seedlings (Fig. 6b, c). Hypocotyl elongation was reduced in double *gid1a-1/gid1b-1* mutant seedlings demonstrating that GA_4_- and DHGA_12_-stimulated hypocotyl elongation is, at least in part, GID1-dependent (Fig. 6b, c). In addition, the fact that DHGA_12_-enhanced hypocotyl elongation is only partially inhibited in *gid1a-1/1b-1* mutants implies that besides GID1a and GID1b, the effect of DHGA_12_ may be mediated by the remaining GID1c homolog.

### GAS2 alters the ABA/GA ratio during seedling development

**Fig. 7:**
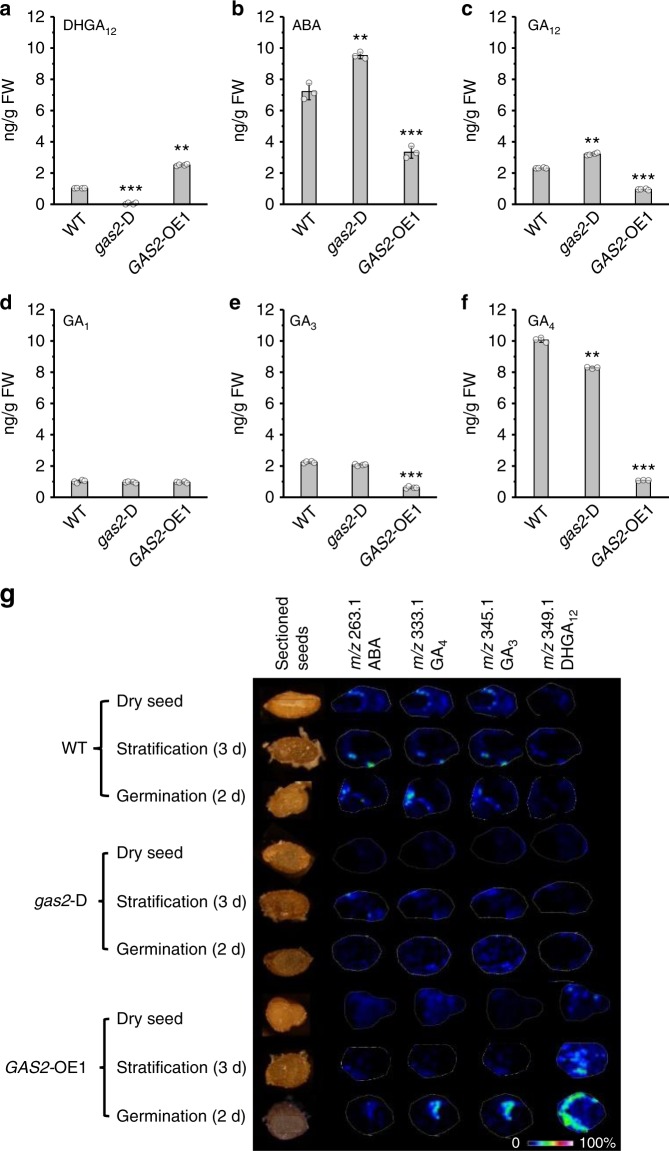
Endogenous GAs and ABA levels in seeds. **a**–**f** DHGA_12_, ABA, GA_12_, GA_1_, GA_3_ and GA_4_ levels in wild-type, noninduced *gas2*-D and *GAS2*-OE1 seeds. 600 mg *Arabidopsis* seeds imbibed in 4 °C overnight were used for each sample. Values are expressed as means ± SD (standard deviations) (*n* = 3), **P < 0.01, ***P < 0.001, *t* test versus WT. **g** Visualization of ABA, GA_3_, GA_4_ and DHGA_12_ in wild-type, noninduced *gas2*-D and *GAS2*-OE1 seeds by MALDI-TOF imaging. Representative images of >3 measurements are presented. Source data are provided as a Source Data file. GA gibberellins, ABA abscisic acid

In order to evaluate the ABA/GA balance in different *GAS2* genetic backgrounds, WT, noninduced *gas2-*D and *GAS2-*OE1 seeds were collected for quantification of endogenous DHGA_12_, ABA, GA_12_, GA_1_, GA_3_ and GA_4_ levels using HPLC-MS/MS (Fig. 7a–f and Supplementary Fig. 23). DHGA_12_ levels were virtually undetectable in noninduced *gas2*-D samples, but were clearly detectable in WT and experienced a sharp rise in *GAS2-*OE1 seeds (imbibed in 4 °C overnight) (Fig. 7a and Supplementary Fig. 23a–c). In contrast, ABA levels in *GAS2-*OE1 seeds (imbibed in 4 °C overnight) were ~50% lower than that in the WT, whereas noninduced *gas2*-D seeds showed higher levels than that in the WT (Fig. 7b and Supplementary Fig. 23d–f). Furthermore, we found that expression levels for several genes involved in the ABA synthesis pathway (e.g. *NCED*3, *ABA1*) were altered in *gas2*-D and *gas2*-D plants following induction with E2 (Supplementary Fig. 24a). Compared with WT, GA_12_ levels were enhanced in noninduced *gas2-*D and reduced in *GAS2-*OE1 seeds (Fig. 7c and Supplementary Fig. 23g–i), consistent with our hypothesis that GAS2 metabolizes GA_12_ to produce DHGA_12_ as a branch of the GA_4_ biosynthetic pathway (Fig. 4g). The GA_3_ and GA_4_ levels showed a strong reduction in *GAS2-*OE1 seeds, perhaps reflecting the observed decrease in GA_12_ levels, which is consistent with the severe reduction in *GA20ox1* expression observed in *GAS2*-OE1 plants (Supplementary Fig. 24b) and/or an increase in the conversion of GA_12_ to DHGA_12_. GA_1_ levels were not altered in any of the studied genotypes (Fig. 7d–f and Supplementary Fig. 23j–r). We also used imaging mass spectrometry (IMS) to investigate the possible correlation between the *GAS2* expression levels and the endogenous GA and ABA levels (Fig. 7g). Dry seeds, imbibed seeds (3 days at 4 °C, and germinating seeds (3 days at 4 °C + 2 days at 22 °C) of WT, noninduced *gas2*-D, and *GAS2*-OE1 lines were analyzed to visualize the endogenous amounts of DHGA_12_, GA_3_, GA_4_ and ABA in the tissue. DHGA_12_ was not detected in dry seeds of WT, consistent with the lack of *GAS2* expression in seeds (Supplementary Figs. 4, 5). Increased DHGA_12_ levels were observed in imbibed and germinating *GAS2*-OE1 seeds compared with WT, whereas the ABA levels appeared to be reduced in *GAS2*-OE1 (Fig. 7g, Supplementary Fig. 25 and Supplementary Table 1). Taken together, the IMS and HPLC-MS/MS data demonstrated a clear correlation between the ABA/GA ratio upon *GAS2* overexpression during dormancy breaking, germination, and early seedling development.

## Discussion

In this study, we provide a detailed analysis of the interdependence of the effects of ABA and GA on early development using genetic and biochemical approaches, resulting in the identification of *GAS2*, a GA-biosynthetic gene, the characterization of its enzymatic activity, the chemical structure of its reaction product, and the establishment of a new route for the biosynthesis of a bioactive intermediate in the GA biosynthesis pathway.

The currently accepted paradigm is that the main pathways for the synthesis of bioactive GAs (e.g. GA_1_ and GA_4_) are catalyzed by the GA20ox subfamily^16,17^ and that the highly biologically active GAs are C_19_-GAs. These all possess a 4,10-lactone, a carboxylic acid (−COOH) at C-6, a hydroxyl group at C-3 in β-orientation, and do not have a hydroxyl group at C-2 in β-orientation. We found that GAS2 uses GA_12_ as a substrate to generate a bioactive GA intermediate with a structure different to previously known bioactive GAs. In vitro, GA_12_ is converted by GAS2 into a product with a predicted structure that identifies it as a member of the GA family (DHGA_12_). Unlike most of the known biologically active GAs (GA_1_, GA_3_, GA_4_, and GA_7_), DHGA_12_ is a C_20_-GA, lacking the 4,10-lactone and the β-hydroxyl group (−OH) at C-3^29,30^.

Although there are obvious structural differences between DHGA_12_ and the known bioactive GAs, we were able to show that DHGA_12_ can bind to the GA receptor GID1, albeit with a lower affinity than GA_4_ (Fig. 6a). It has been documented that GID1 receptors evolved from hormone-sensitive lipases through alteration of the substrate-binding pocket to enhance the affinity and specificity for bioactive gibberellins^27^. We have compared our DHGA_12_-GID1a binding simulation results with previous studies^31,32^, and found that most of the nonpolar contacts by which GID1a interacts with GA_4_ also contact the aliphatic rings of DHGA_12_. Some of the interactions mediated by hydrogen bonds in the GID1a-GA_3_ complex, such as Y31, S116, R244, and D245, are also visible in the interaction with DHGA_12_ (Supplementary Fig. 22). The structural differences between DHGA_12_ and other bioactive GAs, such as the lack of the hydroxylated C-3 as well as the lactone ring present in GA_3_ and GA_4_, suggest that the molecular interactions between DHGA_12_ and receptors have diverged from the established one. The discovery of GAS2 and DHGA_12_ highlights the complexity of GA signaling in *Arabidopsis*, as well as the existence of additional branches in established biosynthetic routes. Further structural research is required to elucidate the details of these interaction mechanisms.

Loss-of-function mutations or silencing of *GAS2* leads to ABA hyposensitivity while overexpression leads to hypersensitivity (Figs. 1 and 2). This is accompanied by a change in the relative amounts of GA and ABA in these lines. Exogenous application of DHGA_12_ promotes seed germination, and reverses the FRL-induced inhibition of hypocotyl elongation, albeit to a lesser degree than the bioactive GA_4_. Further work will be needed to determine to what extent these changes are responsible for the observed seedling development phenotypes. These data suggest that DHGA_12_ is a bioactive GA or at least a GA that is physiologically relevant.

The observations that DHGA_12_ is less active as compared to GA_4_, and the partial complementation of the *gas2* knockdown lines by exogenous DHGA_12_ and GA_4_, imply that there may exist additional factors resulting from the participation of GAS2 in other regulatory processes, perhaps involving interaction with other components of the hormonal metabolism or signaling pathways. It is also possible that additional downstream molecules biosynthesized from DHGA_12_ could be the direct cause of our observations. Finally, it cannot be ruled out that GAS2 may have other substrates and products in planta, which possibly contributes to the partial-rescue phenotypes of *GAS2* silencing/overexpression lines.

Interestingly, the GA-deactivating enzyme CYP714A1 in *Arabidopsis*^33^ and ELONGATED UPPERMOST INTERNODE (EUI) in rice which acts via 16α,17-epoxidation of 13-H GAs^34^ could also block the formation of active GAs, such as DHGA_12_, since GAS2 catalyzes GA_12_ hydration to DHGA_12_ at the 16, 17-double bond. These data imply that GAS2 has function antagonistic to CYP714A1 in *Arabidopsis*. Conversion of GA_12_ to DHGA_12_ by GAS2 may therefore represent a GA synthesis/deactivation branch switch for the double bond oxidation. In fact, induction of *GAS2* expression led to an increase in DHGA_12_ levels and a concomitant reduction in ABA levels while simultaneously decreasing ABA sensitivity, favoring germination over dormancy. This observation points to an important role for GAS2 in the regulation of the balance between the biosynthetic and signaling pathways during germination, with a possible feedback mechanism. Indeed, ABA can induce the expression of *GAS2* whereas GA_4_ represses *GAS2* expression (Supplementary Fig. 5).

In accordance with the regulatory role discussed above, we noticed a significant decrease in the concentration of the more active GA_4_ and ABA in *GAS2-*overexpressing plants. Firstly, GA_12_ is converted to GA_4_ through oxidations catalyzed by GA 20-oxidase (GA20ox) and GA 3-oxidase (GA3ox), respectively, whereas we demonstrate that GAS2 also uses GA_12_ as a substrate to generate an atypical bioactive DHGA_12_. We speculate that GAS2 and GA20ox1 compete for the available pool of cellular GA_12_. Secondly, hormonal levels undergo dynamic changes in different tissues and development stages. For example, GA_4_ concentrations in shoot apices are high in young plants before dropping to very low levels after 2−3 weeks^35^. Shoot apical GA_4_ levels also dramatically increase before floral initiation, and continue to rise until reaching ~100 folds increase by day 56. The dynamic changes on GA levels agree with previous data showing that GA_4_ is the active GA in the regulation of *Arabidopsis* shoot growth and floral initiation^11,36,37^. Similarly, a very substantial decrease in the concentration of the more active GA_4_ was found in the seeds of the *GAS2*-overexpression line (Fig. 7d–f and Supplementary Fig. 24j–r). The balance between ABA and GA action serve as the primary determinant of seed dormancy and germination. The relative reduction observed in ABA and GA_4_ in the *GAS2*-overexpersion plants implies the existence of poorly understood mechanisms controlling the GA/ABA balance. In accordance with this, we found that expression of several ABA synthesis pathway genes (e. g. *NCED3*, *ABA1*) was affected in *gas2*-D and *GAS2*-OE lines. Meanwhile qRT-PCR data indicated that the expression of *GA20ox1* was reduced significantly in *GAS2*-OE1 plants, consistent with the reduced GA_4_ levels observed in *GAS2*-OE1 plants (Fig. 7f). It is well established that active GAs play an important role in the control of seed germination, and the *GAS2* overexpression and loss-of-function phenotypes are consistent with the hypothesis that DHGA_12_ is the active GA in the regulation of *Arabidopsis* seedling establishment. Thirdly, the dynamic GA_4_ and DHGA_12_ could result from distinct hormonal as well as tissue-specific regulation.

This study presents genetic and biochemical evidence of the existence of additional, yet undiscovered, bioactive GAs that control plant responses to specific developmental and environmental conditions. One such example is GAS2, catalyzing the synthesis of a bioactive gibberellin (DHGA_12_). Unlike the structures of traditionally known active GAs, DHGA_12_ is a C_20_-GA and lacks the so-called typical 4,10-lactone. Importantly, GAS2-catalyzed hydration of the GA 16, 17-double bond positions this enzyme as a key modulator of the physiological GA/ABA balance, contributing to the control of crucial physiological processes in plants, such as seed dormancy and germination, and the transition from heterotrophic to autotrophic growth.

## Methods

### Plant materials and growth conditions

*Arabidopsis* ecotype Columbia (Col-0) was used for this study. The *gas2*-D mutant was obtained from an estradiol-inducible activation mutant pool and was isolated by screening seeds on MS medium supplemented with 0.5 μM ABA and 5 μM 17 β-estradiol. The T-DNA insertion site was identified by TAIL-PCR. Primer sequences are listed in Supplementary Table 2.

For the production of *GAS2* overexpression plants, the full-length *GAS2* cDNA amplified with the primers of GAS2OE-F and GAS2OE-R was cloned into the binary pBIB vector under the control of the Cauliflower Mosaic Virus 35S promoter. The construct was introduced into *Agrobacterium tumefaciens* strain GV3101, and *Arabidopsis* plants transformed using the floral dip method^38^. Primer sequences are listed in Supplementary Table 2.

Unless otherwise specified, seeds used in seed germination tests were sterilized and kept for 3 days at 4 °C in the dark to break dormancy on different media (MS, MS + E2, MS + ABA, MS + ABA + E2) solidified with 0.6% agar. The plates were then transferred to a culture room at 22 ± 2 °C under a 16 h light/8 h dark photoperiod. Seed germination percentages were scored for three independent biological replicates.

The hypocotyl length test was performed under far-red light. Seeds were sterilized and kept for 4 days at 4 °C in the dark to break dormancy on the different media (MS, MS + 5 μM DHGA_12_, MS + 5 μM GA_4_). We employed an LED light source emitting light (FR: 5 μmol/m^2^/s^2^) with a peak wavelength of 730 nm and a half bandwidth of 25 nm (Quantum Devices). After 5 days, plates were photographed and hypocotyl lengths measured.

For the experiments involving measurement of *GAS2* mRNA levels in response to red and far-red light exposure. *Arabidopsis* seedlings were exposed to the following conditions. T1: Far red light 2 h; T2: Far red light 2 h + red light 2 h; T3: Far red light 2 h + red light 2 h + Far red light 2 h. The intensity of the FR light was 60 μmol/m^2^/s^2^, and the intensity of R light was 60 μmol/m^2^/s^2^. Expression levels were determined by RT-qPCR.

### RNA isolation and expression analyses

RNA was isolated from 100 mg tissue samples using the TRIzol reagent (Invitrogen). Total RNA samples (2 μg) were used for reverse transcription with Moloney Murine Leukaemia Virus reverse transcriptase (Promega). Quantitative RT-PCR was used to measure gene transcript levels. Three biological replicates were performed.

RT-qPCR was performed using a sequence detector system (7500 Fast, Applied Biosystems) with SYBR Green I. The mean value of three biological replicates was normalized using *tubulin* as an internal control. The relative quantification method (ΔΔCT) was used to evaluate the relative differences (fold-changes) in transcript levels^39^. Primer sequences are listed in Supplementary Table 2.

### GAS2 enzymatic activity and product identification

Enzyme assays employed recombinant GAS2 protein, and 10 ng 17-17-[^2^H_2_]-labeled GA_12_, 17-17-[^2^H_2_]-labeled GA_15_, and 17-17-[^2^H_2_]-labeled GA_24_ (purchased from Prof. L. Mander, Australian National University, Australia) as substrates, as described previously^40^. In brief, [^2^H_2_]GA_12_, [^2^H_2_]GA_15_, [^2^H_2_]GA_24_ was added to the reaction mixtures in the presence of 50 mM Tris, pH 7.8, and a cofactor mixture (24 µL, containing 133 mM 2-oxoglutarate, 133 mM ascorbate, 16.7 mM FeSO_4_, and 33.3 mg/mL catalase) in a total volume of 224 µL. Fresh cofactors were added after 2, 4, 6 and 8 h (24, 24, 24 and 104 µL, respectively). Then the reaction mixtures were incubated at 30 °C overnight and extracted three times. The products were analyzed by liquid chromatography/mass spectrometry (LC-MS) (LCQ Deca AMX, HPLC-electrospray ionization (ESI)–MS, Thermo-Finnigan, USA)^41^. MS-MS data were analyzed using Xcalibur 2.1 software (Thermo-Finnigan). The retention times of samples were compared to deuterium-labeled GA standards.

### Microscale thermophoresis (MST)

Fluorescent labeling was performed using reactive RED dye (NT-647) following the manufacturer’s protocol (Nanotemper, Germany). The labeling procedures were optimized for the GID1c protein to give about 2 tracer molecules per protein, as described by the manufacturer (Nanotemper, Germany). Free dye was removed by Sephadex G-25 column chromatography. MST assays were carried out as described previously^42^, except that serial dilutions of unlabeled GA_4_, GA_12_, and DHGA_12_ were respectively mixed with 200 nM of NT-647-labeled proteins in buffers containing 20 mM Tris-HCl (pH 8.0), 200 mM NaCl, and 0.05% Tween-20. MST data were analyzed using the Hill equation.

### Molecular docking simulations

To examine the interaction of GID1a and DHGA_12_, a model of GID1a was obtained from the X-ray crystallographic structure of GID1a^32^, as downloaded from the RCSB Protein Data Bank (2ZSH) at a resolution of 1.80 Å. GA_3_ and water molecules were removed from the protein structure for the docking simulations; the protein was regarded as ligand-free. Docking simulations were performed using Autodock 4.2 with AutoDockTools^43–45^. The GID1a grid box was set according to the similar part of the GA_3_ binding pocket in the GID1a-GA_3_ complex^32^. The number of 20 modes was selected for each docking run. Other parameters were set to their default values. The pose with lowest energy of binding or binding affinity was extracted and the best binding energy was acquired.

### MALDI-FTICR MS analysis

For GA_12_ and DHGA_12_ detection, cell lysates were extracted from protoplasts transiently transformed with *35S::GAS2-GFP* or *35S::GFP*, and from protoplasts prepared from transgenic plants overexpressing *35S::GAS2*. The GA_12_ substrate (OlChemIm, Czech Republic) was incubated with cell lysate in a total reaction volume of 4 mL, at 22 °C for 16–20 h. The reaction mixture was then broken by addition of 100 μL 90% MeOH, followed by incubation at 4 °C overnight. MALDI-FTICR MS analysis was performed on the supernatant fractions, using a dried-droplet sample preparation protocol: 1 μL of sample solution was mixed with 1 μL of matrix solution, and 1 μL mixture was then pipetted onto the stainless steel target probe, followed by drying under a stream of nitrogen gas at room temperature. A 9.4T Solari X MALDI-FTICR MS (Bruker) equipped with a SmartBeam Nd: YAG 355 nm laser was utilized. The laser was fired at a repetition rate of 2000 Hz. The negative-ion mass spectra in reflectron mode were collected with a pulsed ion extraction time of 200 ns, an accelerating voltage of 19.0 kV, an extraction voltage of 16.6 kV, a lens voltage of 8.0 kV, and a reflector voltage of 21.0 kV. The mass spectra data were acquired over a mass range of *m/z* 200–600 Da with a resolving power of 1000 Hz (using a 6.71 s time-domain transient length) and visualized using Compass Data Analysis 5.0 (Bruker Daltonics, Billerica, MA)^46^.

### GA and ABA measurement

Six hundred milligrams of dry seeds was used for GA and ABA measurement following previous methods with slight modifications^47^. Six hundred milligrams of dry seeds was imbibed at 4 °C overnight. Samples were then frozen and were ground in liquid nitrogen using a mixer mill MM400 (RetschGmbH, Haan, Germany) in 2 mL Eppendorf tubes. The resultant powder was extracted with 1 mL of extraction solvent (methanol: H_2_O, 90:10 (v/v)) using ultrasonication (4–7 °C). The labeled forms of the compounds d6-ABA, d2-GA_1_, d2-GA_3_, d2-GA_4_, and d2-GA_12_ were added as internal standards. After centrifugation (10,000 × *g* for 15 min at 4 °C), the supernatant was collected, the pellet was re-extracted with 0.5 mL of extraction solvent, and the extraction repeated three times. The supernatants were combined and dried thoroughly under a nitrogen stream, then re-dissolved in 300 μL of methanol before being subjected to centrifugation (10,000 × *g* for 5 min at 4 °C) and filtration through a 0.22 μm PTFE filter (Waters, Milford, MA, USA). Samples (5 μL) were analyzed using liquid chromatography/mass spectrometry (LCQ Deca AMX, high-performance liquid chromatography (HPLC)-electrospray ionization (ESI)-MS, AB SCIEX-4000 QTRAP, USA). Hormones were measured from two independent samples for each treatment^48,49^.

Quantification was performed using calibration curves including each of the five unlabeled analytes (ABA, GA_1_, GA_3_, GA_4_, and GA_12_). Quantitative analysis of GA and ABA by HPLC-MS/MS was performed using ^2^H-labeled GAs and d6-ABA as internal standards^38,39^. As commercial DHGA_12_ is unavailable, DHGA_12_ quantification was performed using [^2^H_2_] GA_12_ as an internal standard. GA and ABA levels were determined in triplicate, independent seed samples, by liquid chromatography tandem mass spectrometry (LC-MS)^50^.

### Chemical reaction and structure identification

GA_12_ (4 mg) was dissolved in 4.0 mL methanol by ultrasonication and transferred to a reaction flask. Subsequently, 500 µL H_3_PO_4_ (1.0 mol/L) and 3.5 mL water were added to the flask followed by stirring at 700 rpm on a water bath at 60 °C for 8 h. The mixture was separated and detected by LC-MS in negative mode with a full scan from 100 to 500 Da. The structure of DHGA_12C_ was identified using Waters MassLynx 4.1 software of Waters ACQUITY UPLC H-Class and Waters SYNAPT G2-Si HDMS.

### MALDI-TOF MS

Samples comprising 11 seeds were selected from dry, vernalized, and germinating *Arabidopsis* seeds; these seed samples were treated as three biological replicates with separate sample preparation and measurement. Measurements were performed using a Time-of-Flight mass spectrometer (Bruker Daltonics, Autoflex Speed) in reflectron mode. The instrument was equipped with a pulsed, 352 nm solid-state laser (Bruker Daltonics, 2 kHz SmartBeam II) operated at a repetition rate of 2000 Hz and a laser pulse energy of 100–190 μJ. The spatial resolution was kept in imaging mode (20 μm), and mass spectra recorded from 500 laser shots for each spot using the default random walk method (Bruker, FlexImaging 4.0). Spectra were recorded in negative-ion mode at 150–400 *m/z* range. The operating voltage conditions in reflection mode were as follows: ion source 1, 18.95 kV; ion source 2, 16.55 kV; lens, 8.01 kV; reflector 1, 21.02 kV; reflector 2, 9.79 kV. The delay time was 200 ns. Seed samples were individually split into halves using a razor blade. The tissue surface was selected for imaging based on a dry appearance with no bright and visible liquid on its surface. Materials were transferred to an ITO glass slide surface, by allowing the two surfaces to touch each other for 1 s, followed by sample removal and drying of the target by nitrogen flow. All imaging data were normalized with the total ion chromatogram; the highest normalized value of all MS was set to 100%. Matrix solution NEDC (*N*-(1-naphthyl) ethylenediamine dihydrochloride), in a 3:7 mixture of ethanol and water, was sprayed over the sample using an ImagePrep automatic matrix sprayer (Bruker) until the entire tissue surface was homogeneously covered.

### Generation of CRISPR lines

The CRISPR construct was designed and produced to generate the knockout mutants^51^. To genotype CRISPR-induced mutations, a 538-bp region including the guide RNA site was amplified by PCR and sequenced by Sanger sequencing. T_2_ homozygous mutant plants were obtained and confirmed by sequencing. The sgRNA sequence and genotyping primers for *GAS2* are listed in Supplementary Table 2.

### Subcellular localization

Protoplasts were isolated from *Arabidopsis* leaves and transformed with a GAS2-GFP fusion constructs^52^. Fluorescence was examined using a laser scanning confocal microscope (LSM710, Zeiss, Germany). The protoplasts were excited at 488 nm and fluorescence was detected at 500–550 nm for GFP. The transmission fields were collected simultaneously for use in merged images.

### GUS staining

Transgenic lines containing the *GAS2pro*::*GAS2*-*GUS* constructs were tested for GUS activity by incubation in GUS staining buffer (3 mmol/L 5-bromo-4-chloro-3-indolyl β-glucuronic acid, 0.1 mol/L sodium phosphate buffer, pH 7.0, 0.1% Triton X-100, and 8 mmol/L β-mercaptoethanol) at 37 °C overnight in darkness. Staining was terminated by replacement of the staining solution with 70% ethanol, and the samples were stored at 4 °C until observation under the microscope.

### Phylogenetic analysis

Protein sequences were retrieved from the *Arabidopsis* protein database and searches for similar sequences was performed by BLAST analysis. Phylogenetic trees were generated using MEGA7 software^53^. Statistical support for the nodes on the Neighbor-Joining trees were evaluated by bootstrap analysis.

### Protein alignment

COBALT software^54^ was used to perform multiple alignment of protein sequences using default parameters. The Hidden Markov Model (HMM) profiles of the DIOX_N (PF14226) and 2OG-FeII_Oxy (PF03171) domains were downloaded from the pfam website (http://pfam.xfam.org/). The positions of the DIOX_N and 2OG-FeII_Oxy domains were determined by using HMMSEARCH^55^.

### DHGA_12_ measurement

To further investigate whether GA_12_ is an endogenous substrate for GAS2, the GAS2-GFP fusion (*35S::GAS2-GFP*) and the *35S::GFP* control were respectively infiltrated into tobacco leaves for agrobacterium-mediated transformation. Following infiltration, these transformed tobacco plants were cultured at 22 °C for 16–20 h under continuous light conditions for 3 days^56^ and protoplasts were then isolated from the leaves^57^. The detached protoplasts from these samples were treated with 2.5 µg/mL overnight to detect the GA intermediate. The reaction mixture was then broken by addition of 100 μL 90% MeOH. All data were obtained using a Q Exactive™ Hybrid Quadrupole-Orbitrap™ Mass Spectrometer (ThermoFisher) equipped with C18 column (100 cm × 2.1 mm, 1.7 μm), with methanol and water (85/15, v/v) as the mobile phase (0.2 mL/min).

### NMR

The one-dimensional ^1^H and ^13^C NMR spectra were recorded at 298 K on a Bruker 850 MHz spectrometer equipped with a triple resonance 5 mm HCN-cryoprobe. The chemical shifts were referenced to 0.1% internal tetramethylsilane (TMS). The two-dimensional NMR spectra including NOESY, COSY, HMQC, and HMBC were recorded at 298 K on Bruker 600 MHz spectrometer equipped with a triple resonance 5 mm HCN-cryoprobe. All 2D spectra were collected with 256 × 4096 matrix with 32 or 40 transients per t1 increment. NOESY spectra were acquired using mixing times of 1 s. The long-range coupling value for HMBC spectra was set to 8 Hz.

### Reporting summary

Further information on experimental design is available in the Nature Research Reporting Summary linked to this article.

## Supplementary information

Supplementary Information

Reporting Summary

Source Data

## Data Availability

The authors declare that all relevant data supporting the findings of this study are included in the main manuscript file or Supplementary Information or are available from the corresponding author upon reasonable request. The source data of images in Figs. 1a, d, 5a, b, e, 6b and 7g as well as the source data underlying Figs. 1b, c, f, 2a−g, 3e, j, 5c, d, f, 6a, c and 7a–f are provided in the Source Data file. For the source data of the Supplementary Information, the source data of the gels and images in Supplementary Figs. 1a, b, d, e as well as the source data underlying Supplementary Figs. 1f, 2a–d, 21a, b, and 24a, b are also provided in the Source Data file.
