# Supplementary material for: Biosynthesis of DHGA12 and its roles in Arabidopsis seedling establishment
Source: Nat Commun. 2019 Apr 16;10:1768. doi: 10.1038/s41467-019-09467-5 (PMC6467921; doi:10.1038/s41467-019-09467-5)
Supplement: Supplementary file 1 — Supplementary Information [file 41467_2019_9467_MOESM1_ESM.pdf]

# Supplementary Data

## Supplementary Fig. 1

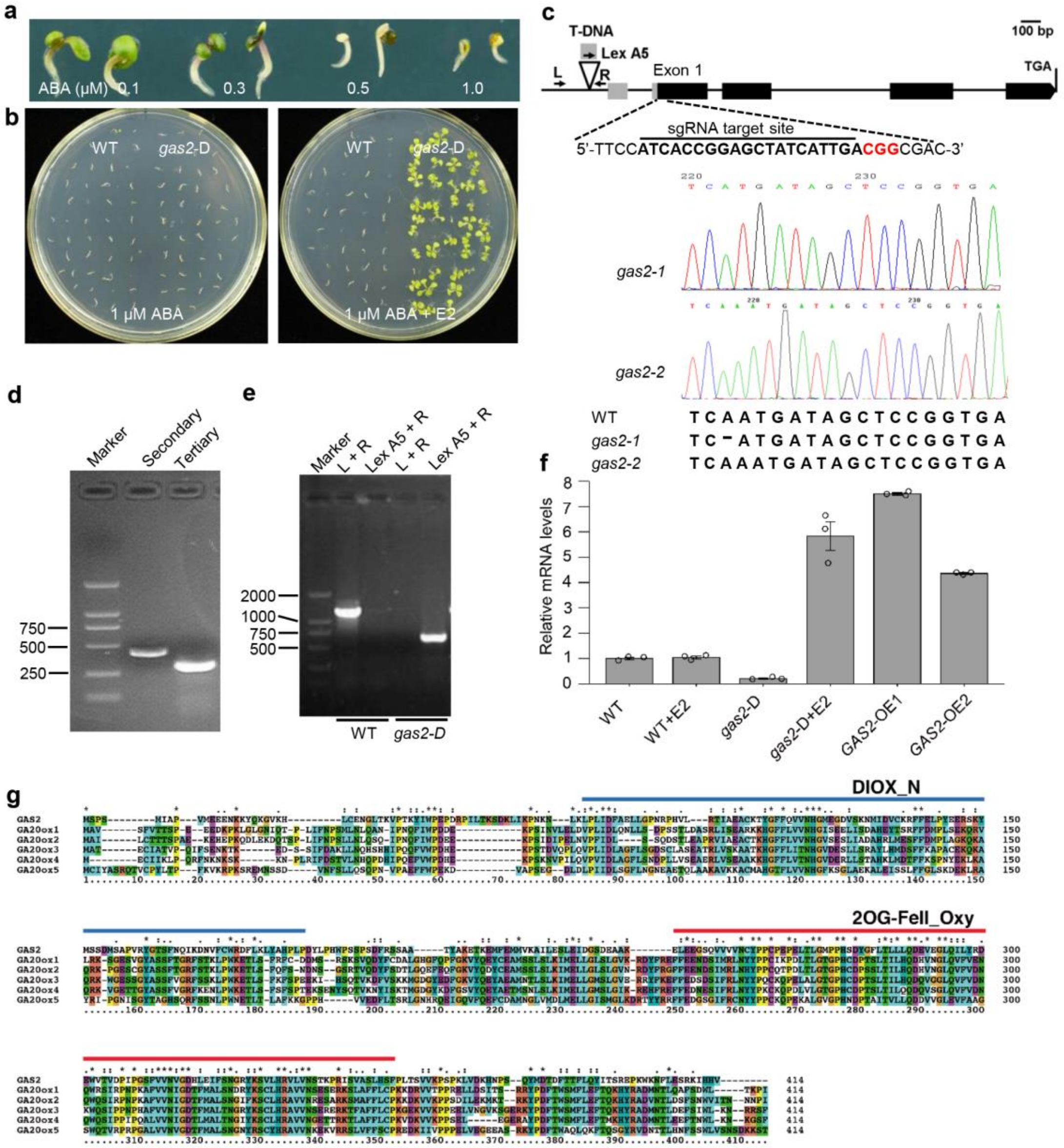


**Supplementary Fig. 1** Isolation of *gas2*-D mutant and generation of *GAS2* CRISPR/Cas9 mutants. **a** Screening system optimization for ABA-insensitive mutants. **b** Phenotypes of 14-d-old WT and *gas2*-D line grown on MS medium supplemented with 1.0 μM ABA (left panel) or 1.0 μM ABA plus 5.0 μM estradiol (right panel). **c** T-DNA insertion site (containing the XVE transactivator) within *GAS2* locus and sgRNA target site in the loss-of-function *gas2* mutants. The sequence of the sgRNA target site in the first exon of *GAS2* gene. The protospacer-adjacent motif

(PAM) sequence highlighted in red letters. Boxes represent exons, including protein-coding regions (black) and untranslated regions (gray). L: forward genomic PCR primer; Lex A5: T-DNA border PCR primer; R: reverse genomic PCR primer. The chromatogram shows the sequencing of the targeted site of the *gas2-1* and *gas2-2* mutants. **d** Agarose gel electrophoresis of secondary and tertiary TAIL-PCR products. The numbers on the left denote the fragment size (bp) of DNA markers. **e** T-DNA insertion verification. PCR products of genomic DNA from WT and *gas2*-D plants using different primer combinations. The numbers on the left denote the fragment size (bp) of DNA markers. **f** *GAS2* mRNA levels of WT and *gas2*-D (with and without E2), *GAS2*-OE1 and *GAS2*-OE2 lines. Error bars represent SD (standard deviation). E2, estradiol. Source data are provided as a Source Data file. **g** Alignment of of GAS2 and GA20ox1-5. Blue line represents the conserved DIOX_N (Non-haem dioxygenase N-terminal domain) domain. Red line represents the conserved 2OG-FeII_Oxy (Oxoglutarate/iron-dependent dioxygenase) domain.

**Supplementary Fig. 2**

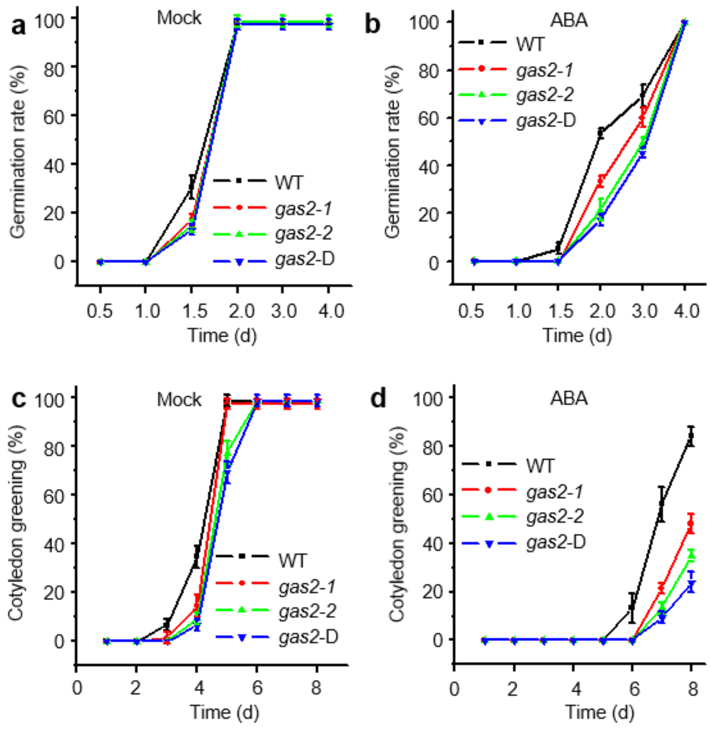


**Supplementary Fig. 2** *GAS2* mutants show hypersensitivity to ABA. **a-b** Gemination rates analysis of seeds of wild type, *gas2-1*, *gas2*-2 and non-induced *gas2*-D grown on MS with or without the addition of 0.2 µM ABA. Error bars represent SD (standard deviations) (n = 72). Source data are provided as a Source Data file. **c-d** Cotyledon greening analysis of seeds of wild type, *gas2-1*, *gas2-2* and non-induced *gas2*-D grown on MS with or without the addition of 0.2 µM ABA. Error bars represent SD (standard deviations) (n = 72). Source data are provided as a Source Data file.

**Supplementary Fig. 3**

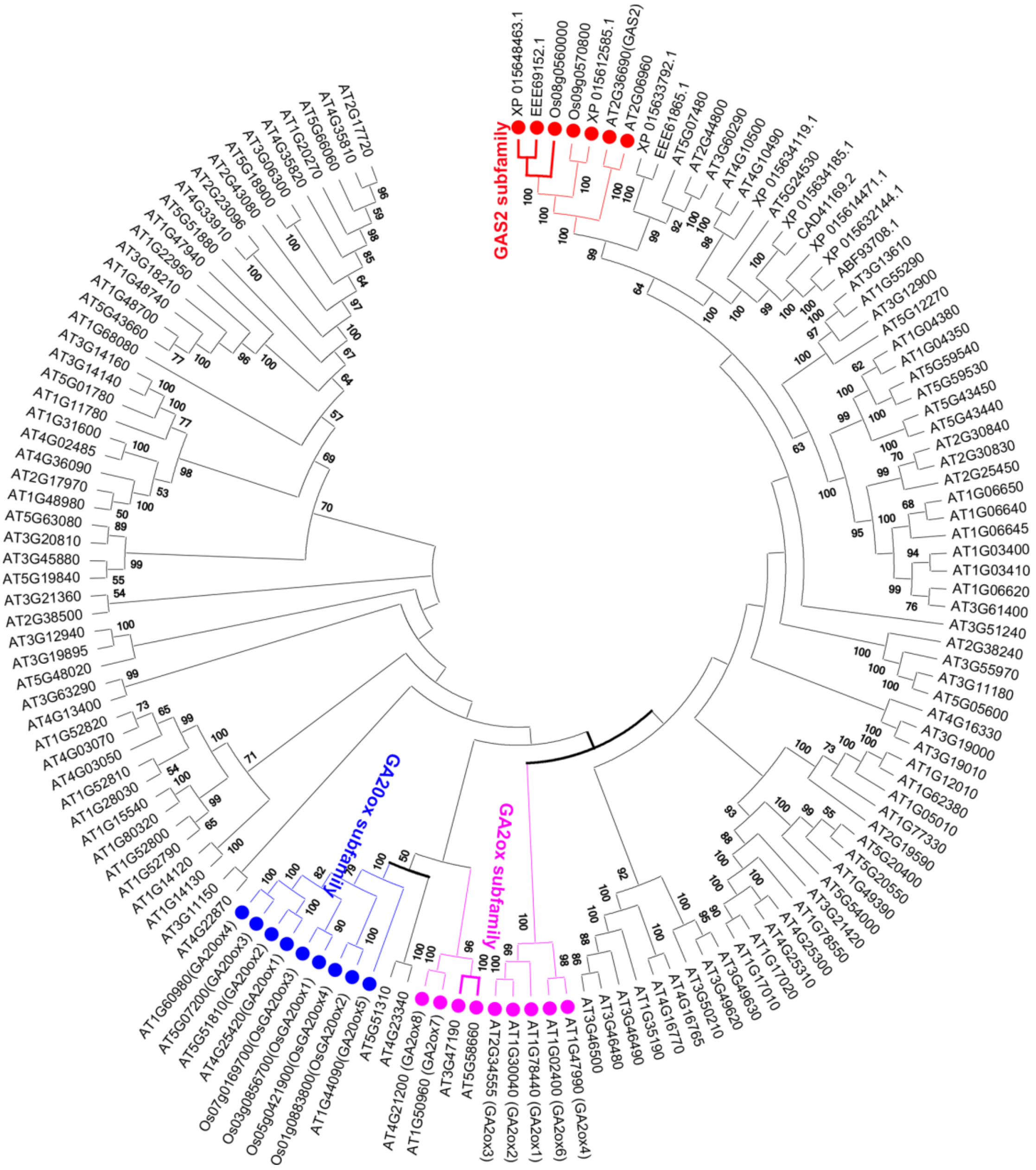


**Supplementary Fig. 3.** Phylogenetic relationship among proteins from the 2-oxoglutarate (2OG) and Fe-dependent oxygenase superfamily in Arabidopsis and rice. The neighbor joining tree includes 126 proteins from Arabidopsis and 17 from rice. Red lines, green lines and blue lines represent the GAS2, GA2ox and GA20ox subfamilies, respectively.

**Supplementary Fig. 4**

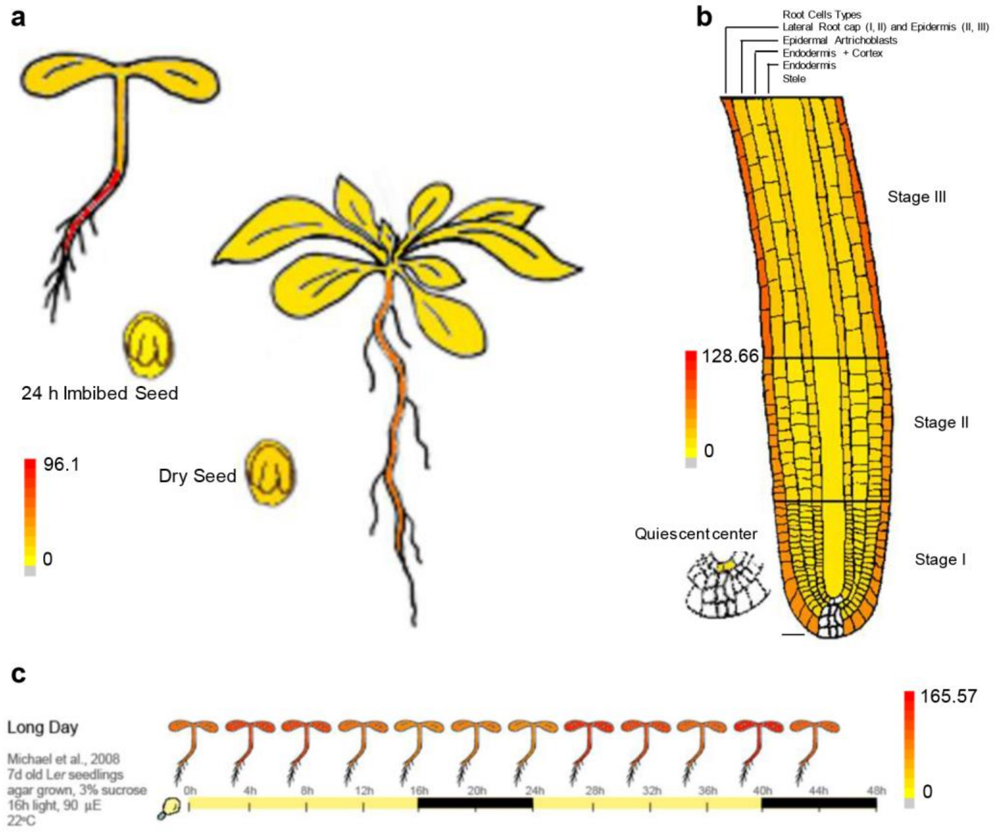


**Supplementary Fig. 4** *GAS2* expression patterns in different tissues and seedlings under 16 h/8 h (light/dark) photoperiod. **a-b** Relative *GAS2* expression levels in seedlings, seeds, roots. Based on microarray data displayed in the eFP Browser (http://www.bar.utoronto.ca/efp/cgi-bin/efpWeb.cgi). **c** Relative *GAS2* expression levels in 7-day-old Ler Seedlings under long day conditions (16 h light/8 h darkness) Based on microarray data displayed in the eFP browser (http://www.bar.utoronto.ca/efp/cgi-bin/efpWeb.cgi). Color scale shows microarray signal level.

**Supplementary Fig. 5**

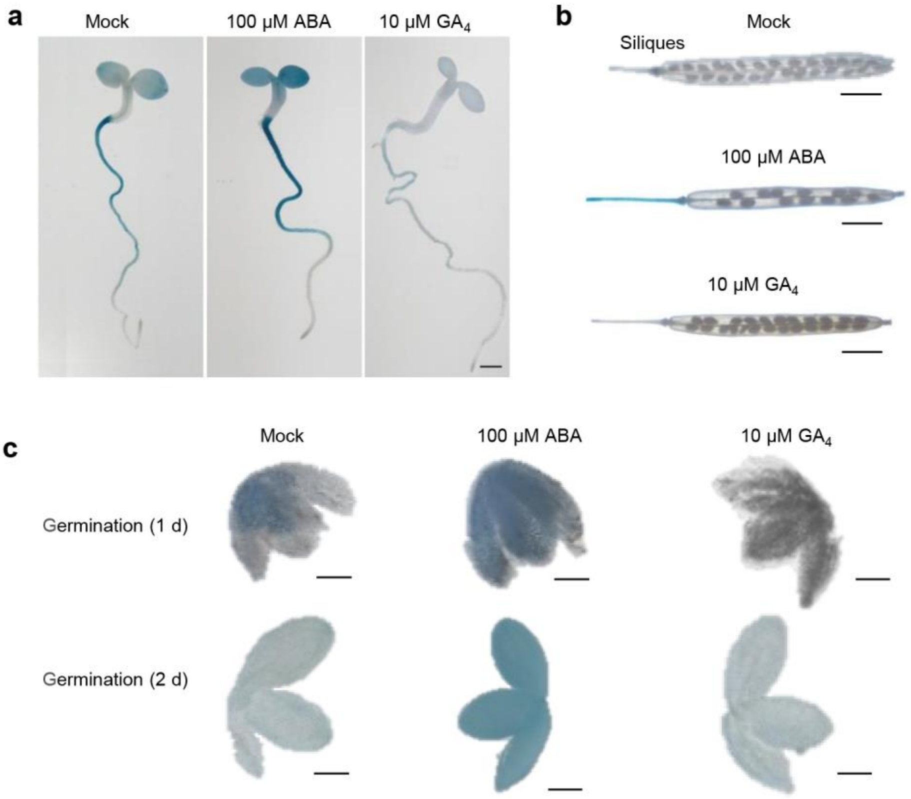


**Supplementary Fig. 5** *GAS2* expression patterns. **a**-**c** GUS staining of 10-d-old seedlings (**a**), siliques (**b**) and 1- or 2-d after germination (**c**) of *GAS2pro*::*GAS2*:*GUS* transgenic *Arabidopsis* lines with or without treatment with 100 µM ABA or 10 µM $GA_4$. Bars = 1 cm in (**a**) and (**c**), 2 cm in (**b**).

**Supplementary Fig. 6**

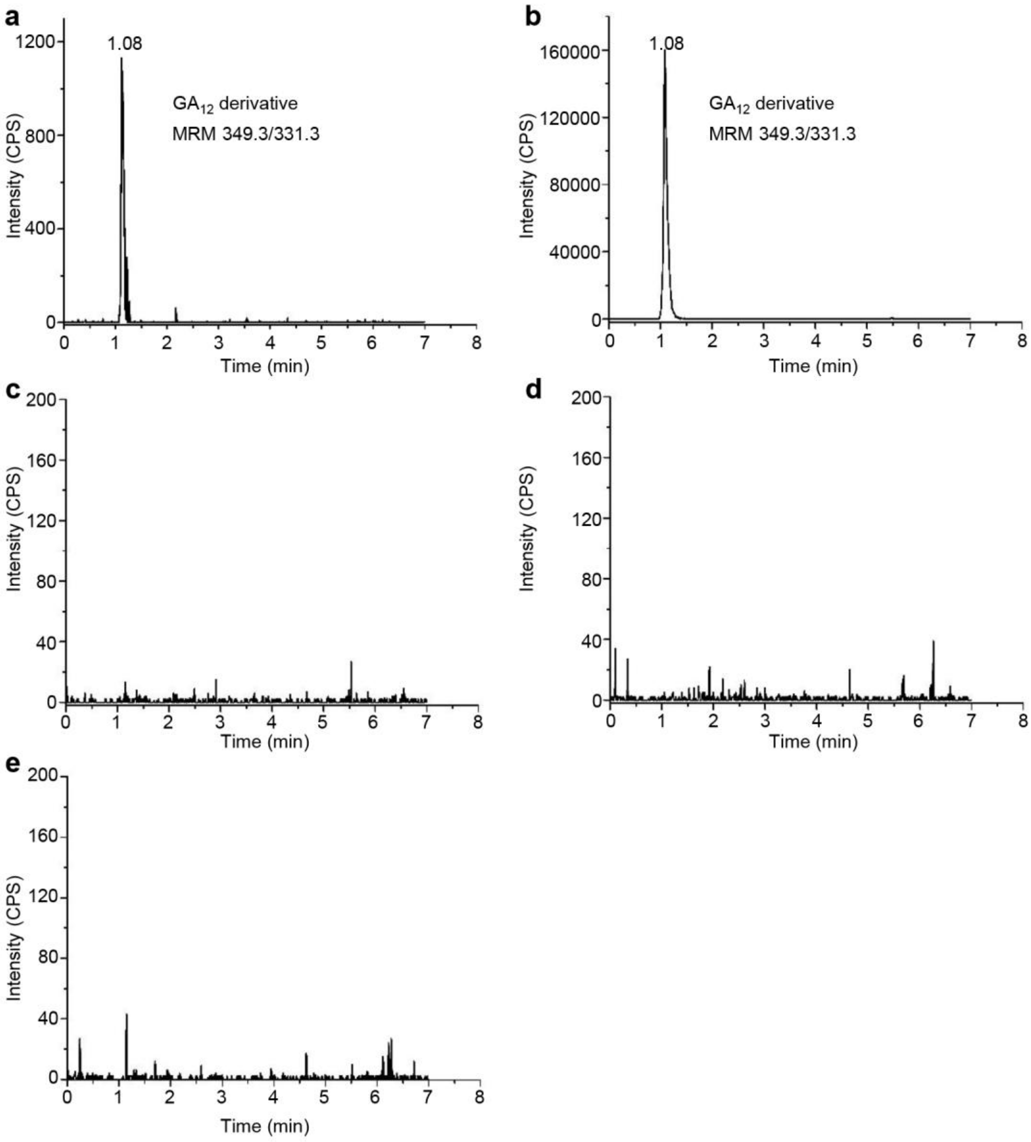


**Supplementary Fig. 6** Analysis of GAS2 catalyzed *in vitro* reaction products using $GA_{12}$ as substrate and recombinant GAS2 using ACQUITY UPLC I-Class Xevo TQS. **a** LC-MS chromatogram of the products produced by the catalytic conversion of $GA_{12}$ by GAS2. **b** LC-MS chromatogram of chemically synthesized $GA_{12}$ derivative. **c** LC-MS chromatogram the reaction in (a) containing denatured GAS2. **d** LC-MS chromatogram the reaction in (a) in the absence of cofactors. **e** LC-MS chromatogram the reaction in (a) in the presence of Fe chelator (EDTA) with a of $Fe^{2+}$ : EDTA ratio of 1:1.

**Supplementary Fig. 7**

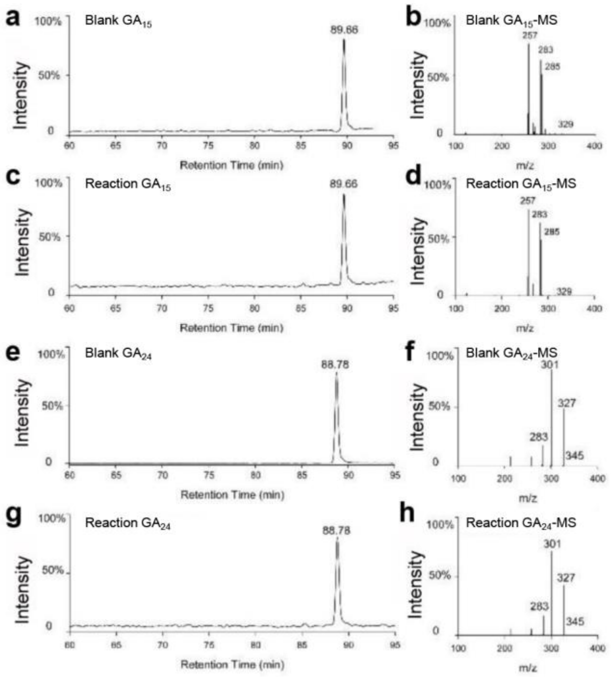


**Supplementary Fig. 7** Analysis of GAS2 activities using $GA_{15}$ and $GA_{24}$ as substrates. LC-MS was run on 24 hours reaction mixes. **a-d** Liquid chromatography chromatogram and mass spectrometry of the reaction mixes of GAS2 catalyzed products with $GA_{15}$ as substrate. **e-h** Liquid chromatography chromatogram and mass spectrometry of the reaction mixes of GAS2 catalyzed products with $GA_{24}$ as substrate.

**Supplementary Fig. 8**

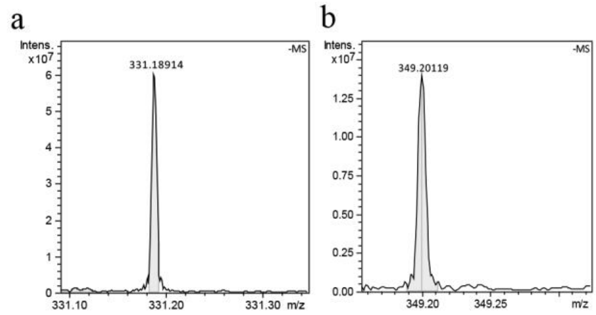


**Supplementary Fig. 8** Mass spectra for the [M-1]$^-$ ions for $GA_{12}$ (**a**) and $GA_{12}$ derivative (**b**) obtained by MALDI-FTICR-MS in negative mode from standard $GA_{12}$ compound and synthesized $GA_{12}$ derivative compound, respectively.

**Supplementary Fig. 9**

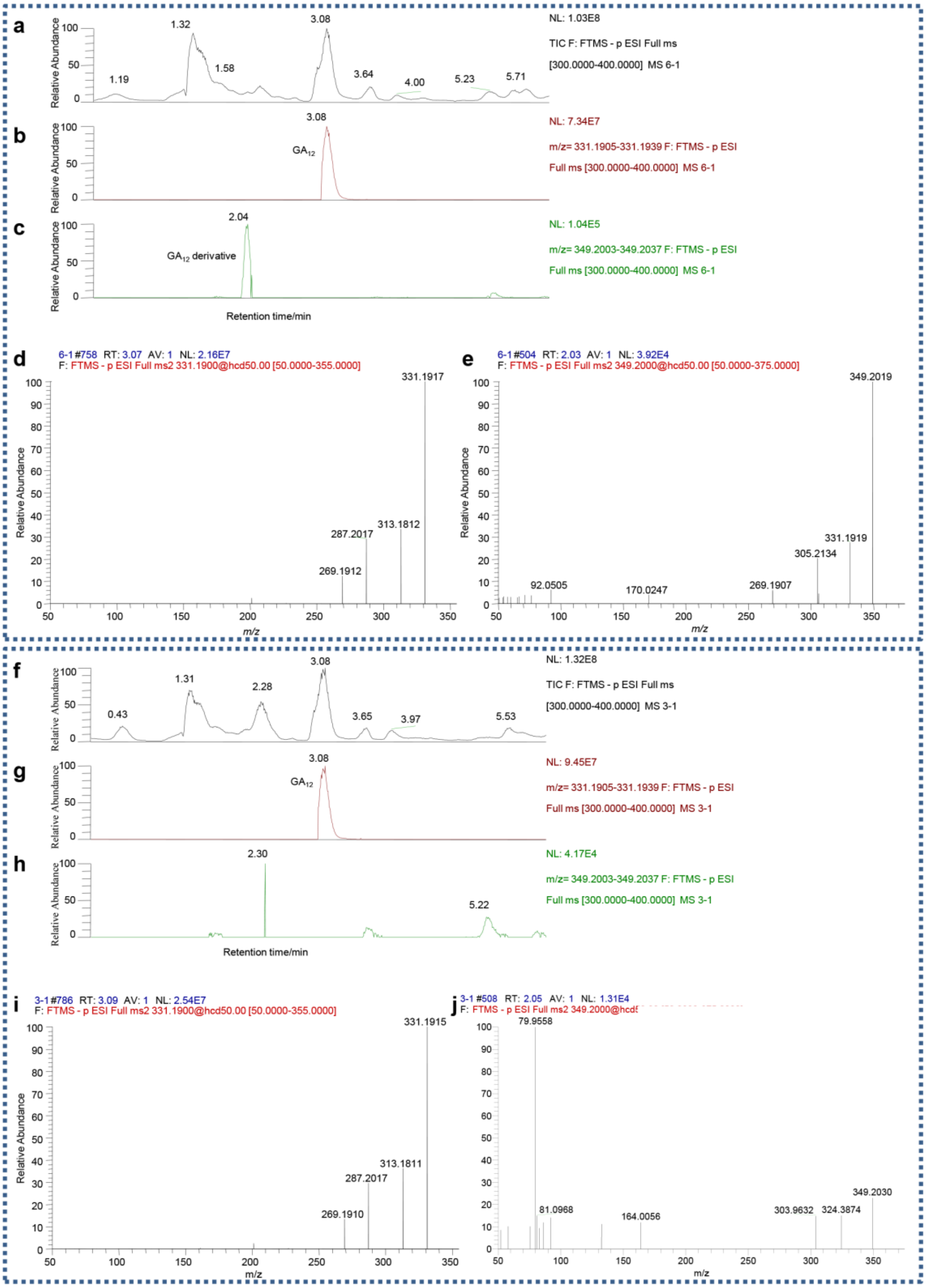


**Supplementary Fig. 9.** LC-MS analysis of $GA_{12}$ derivative formation in protoplasts prepared from tobacco leaves transiently expressing GAS2-GFP fusion protein driven by the *35S* promoter incubated with $GA_{12}$ (**a-e**). Expression of GFP from the same

promoter was used as a negative control (**f-j**). Shown are the total ion chromatograms (for masses from *m/z* 300-400)(**a** and **f**), extract ion chromatograms for *m/z* 331.19 (for $GA_{12}$)(**b** and **g**) and *m/z* 349.20 (for $GA_{12}$ derivative)(**c** and **h**) and MS/MS spectra from the ions at *m/z* 331.19 (**d** and **i**) and 349.20 (**e** and **j**).

**Supplementary Fig. 10**

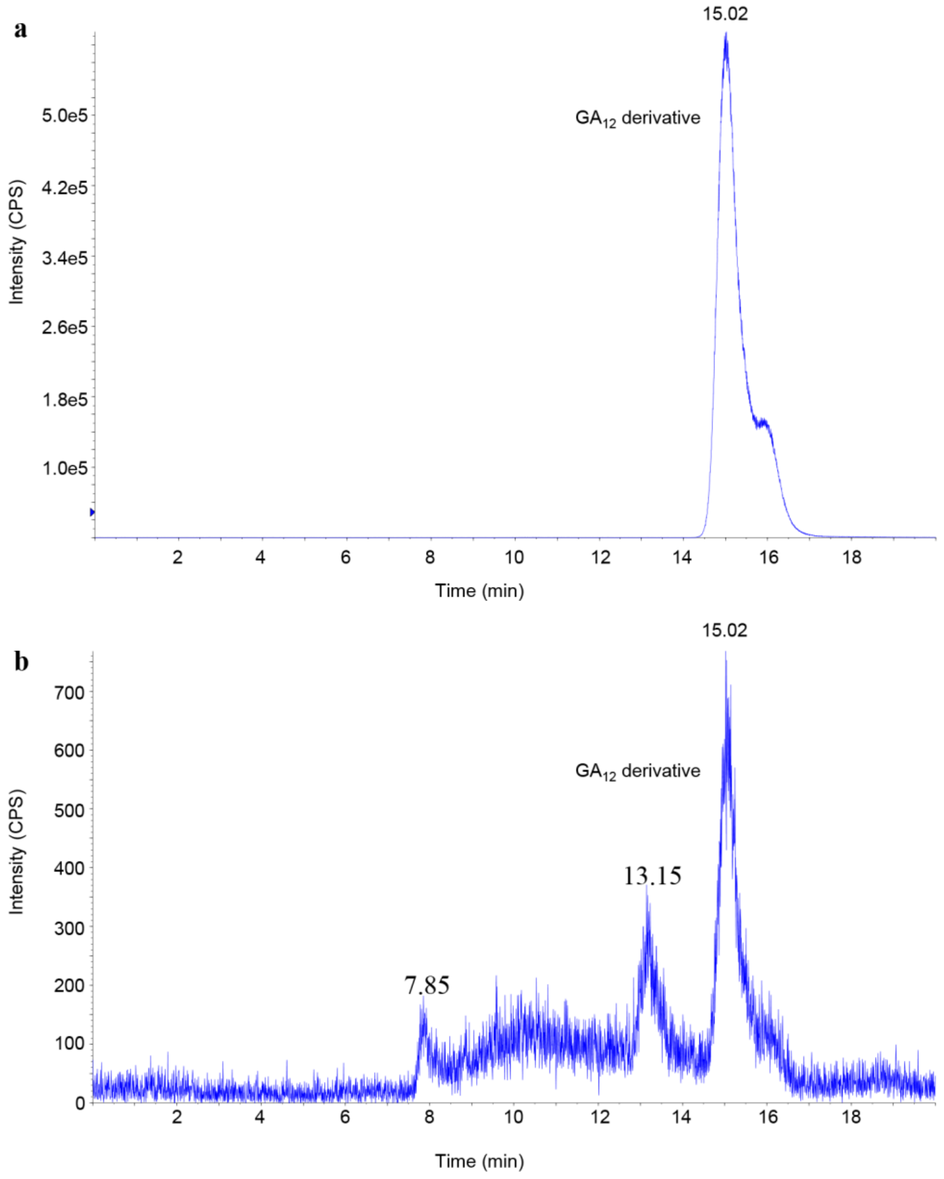


**Supplementary Fig. 10** Chromatograms from UPLC-ESI-MRM (349.3/331.1) for $GA_{12}$ derivative from incubation of $GA_{12}$ with recombinant GAS2 (**a**) and an extract of maize (*Zea mays L.*) seedlings (**b**).

**Supplementary Fig. 11**

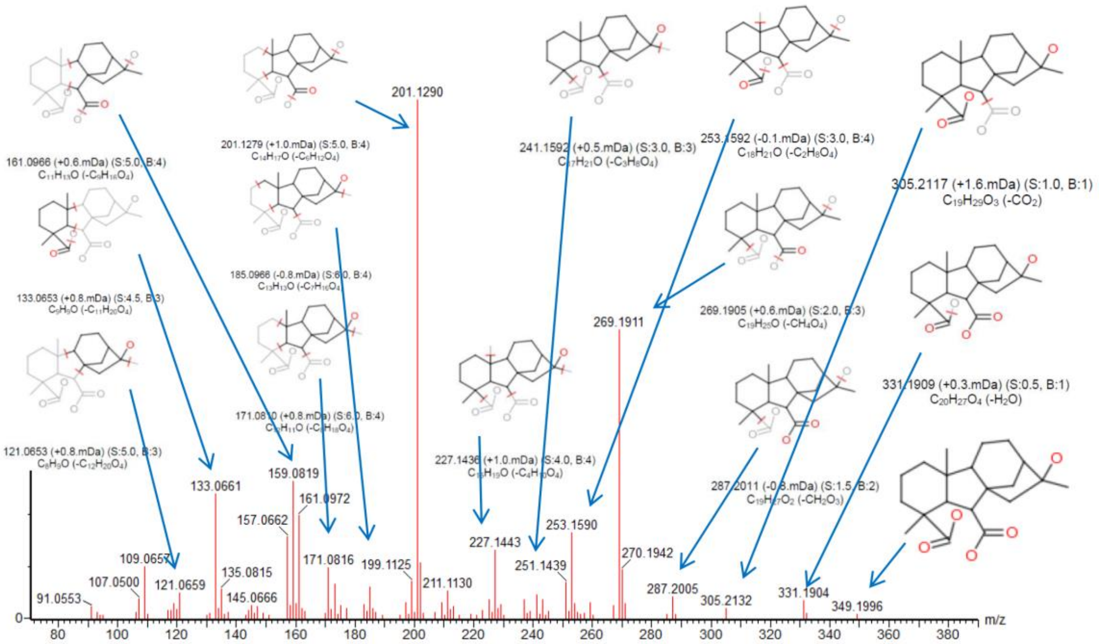


**Supplementary Fig. 11** High resolution MS spectrum of $DHGA_{12}$ and the proposed molecular structures of fragments. The spectrum obtained using the Waters Mssslynx 4.1 software.

**Supplementary Fig. 12**

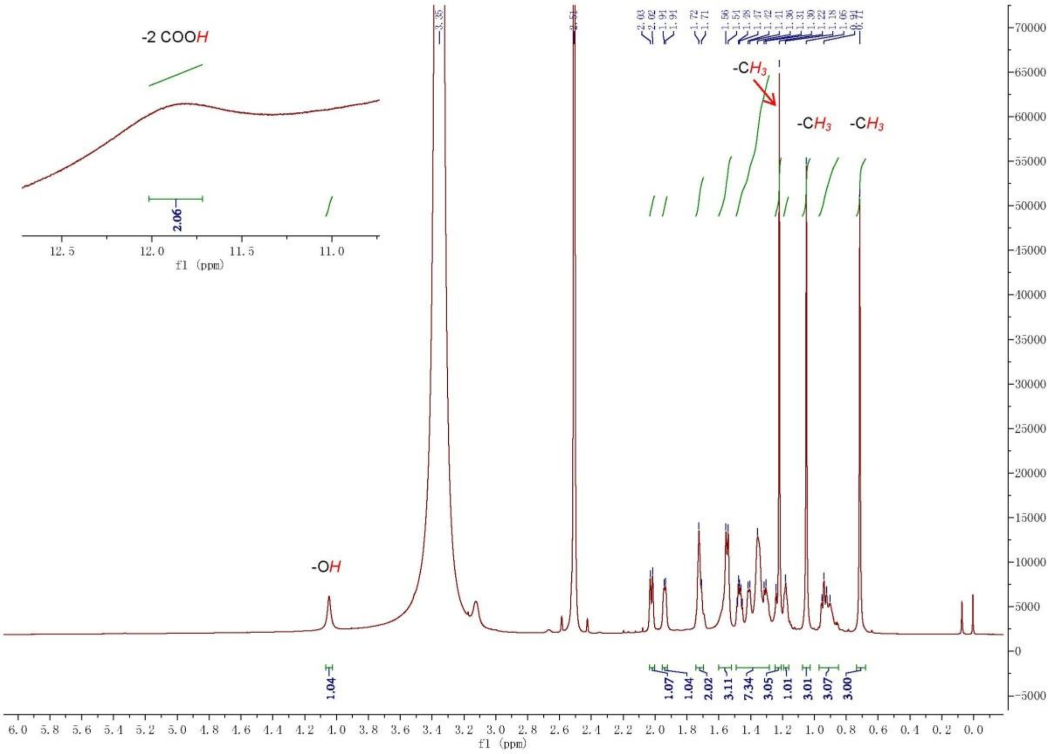


**Supplementary Fig. 12** Proton Nuclear Magnetic Resonance ($^{1}$H-NMR) spectra of $DHGA_{12}$ (850 MHz, $d_6$-DMSO) demonstrating the presences of -*COOH* type, -*OH* type and -*CH3* type protons. δ 4.05 (s, 1H), 2.01 (t, J = 17.4 Hz, 1H), 1.94 (d, J = 6.5 Hz, 1H), 1.76 –1.69 (m, 2H), 1.55 (d, J = 12.1 Hz, 3H), 1.49–1.43 (m, 2H), 1.43–1.38 (m, 1H), 1.38–1.28 (m, 4H), 1.23 (d, J = 17.0 Hz, 3H), 1.20–1.14 (m, 1H), 1.05 (s, 3H), 0.91 (ddd, J = 42.7, 20.9, 11.3 Hz, 3H), 0.71 (s, 3H).

**Supplementary Fig. 13**

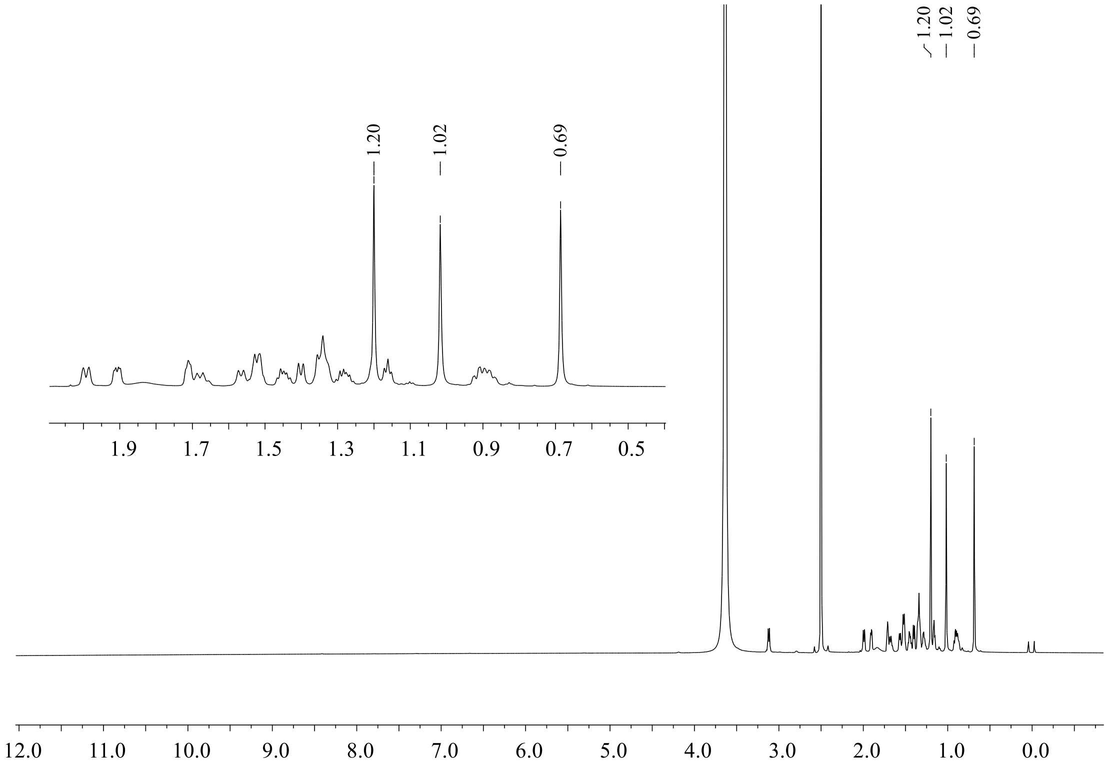


**Supplementary Fig. 13** Proton Nuclear Magnetic Resonance ($^1$H-NMR, 850 MHz) spectra of $DHGA_{12}$ using $d_6$-DMSO/$D_2O$ as solvent. Deuterium experiment: $^1$H NMR of $DHGA_{12}$ was performed in the mixed solvent of $d_6$-DMSO/$D_2O$(note that the peak of -*OH* and -*COOH* disappeared) confirming the presences of -*OH* and -*COOH* type protons.

**Supplementary Fig. 14**

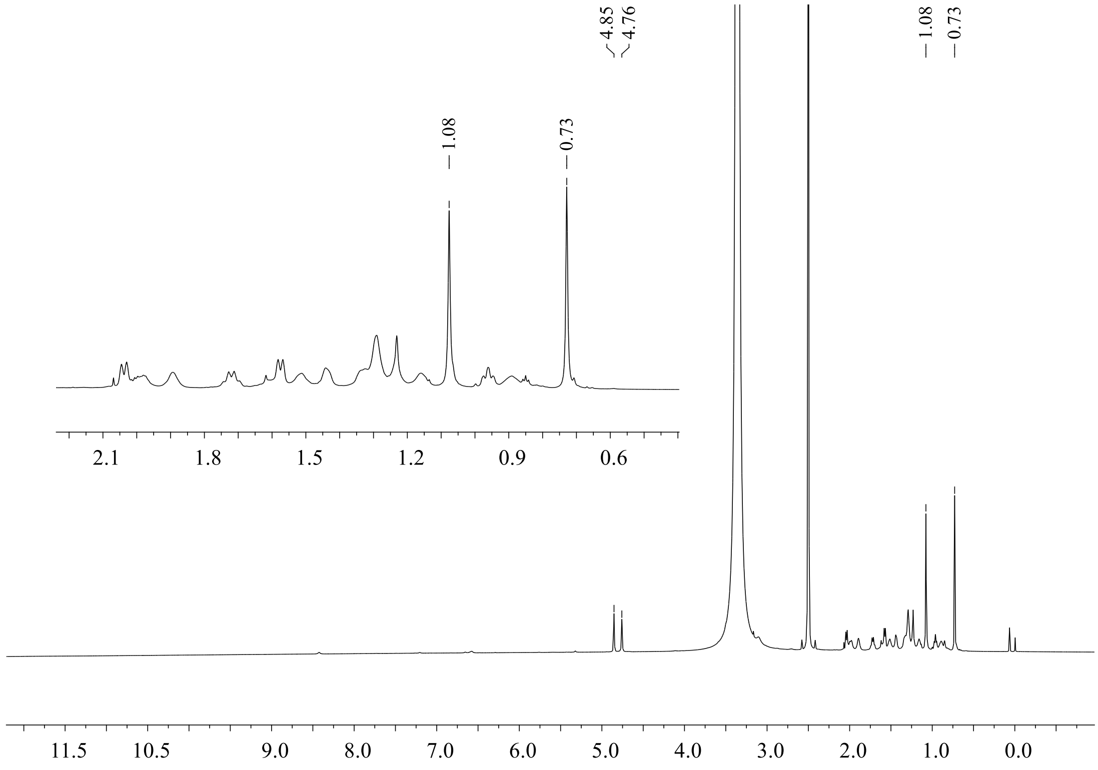


**Supplementary Fig. 14** Proton Nuclear Magnetic Resonance ($^{1}$H-NMR, 850 MHz) spectra of GA$_{12}$ using *$d_6$*-DMSO as solvent.

**Supplementary Fig. 15**

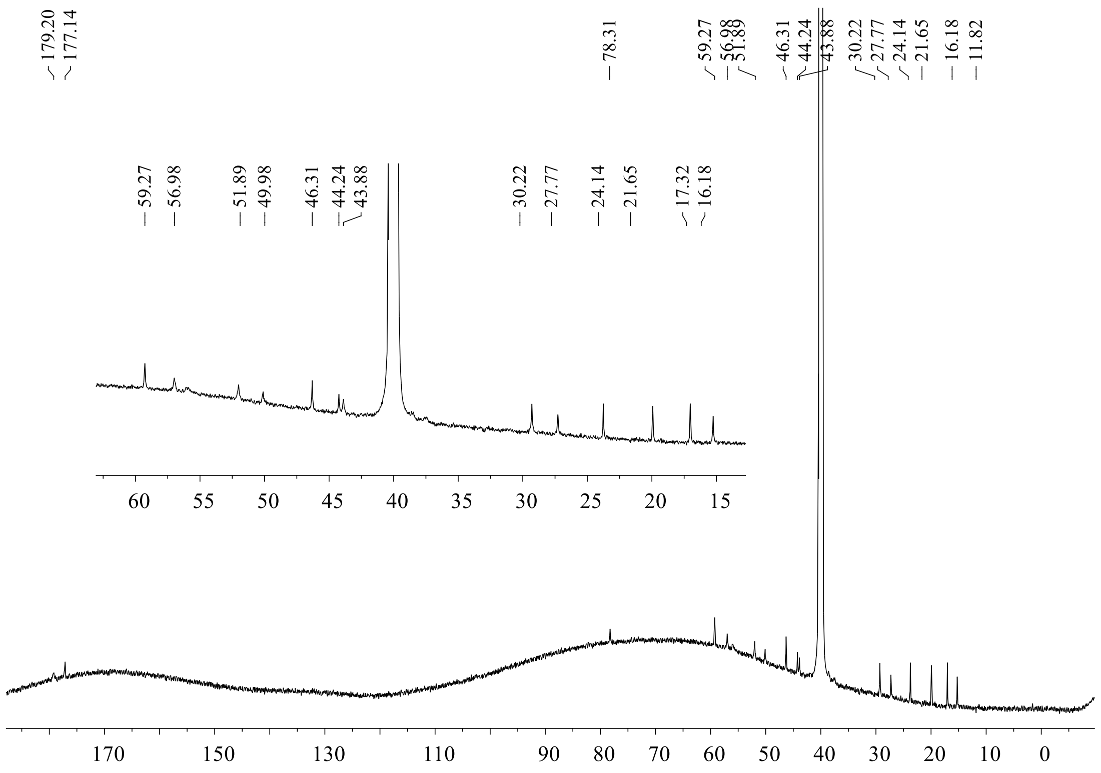


**Supplementary Fig. 15** Carbon Nuclear Magnetic Resonance ($^{13}C$ NMR, 850 MHz) of $GA_{12}$ using $d_6$-DMSO as solvent.

**Supplementary Fig. 16**

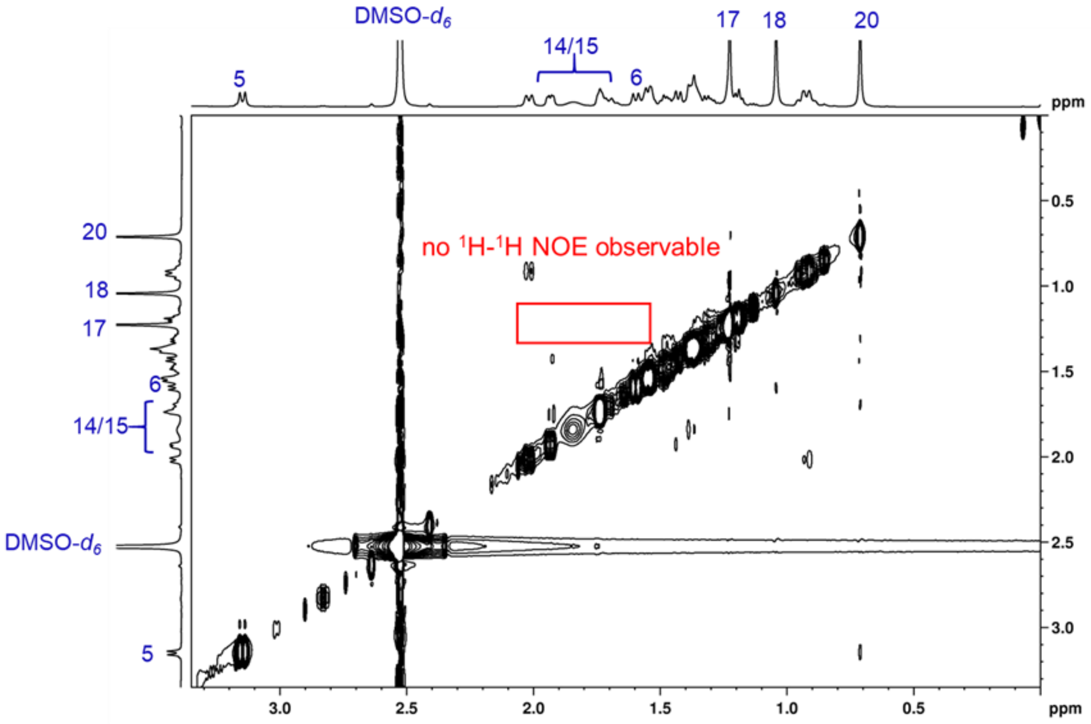


**Supplementary Fig. 16** Proton–proton Overhauser NMR spectroscopy ($^{1}$H-$^{1}$H NOESY spectrum, 600 MHz) of DHGA$_{12}$ in the mixture of DMSO-$d_6$/$D_2O$ confirming the chirality of the -$CH_3$ at 17 position of DHGA$_{12}$.

**Supplementary Fig. 17**

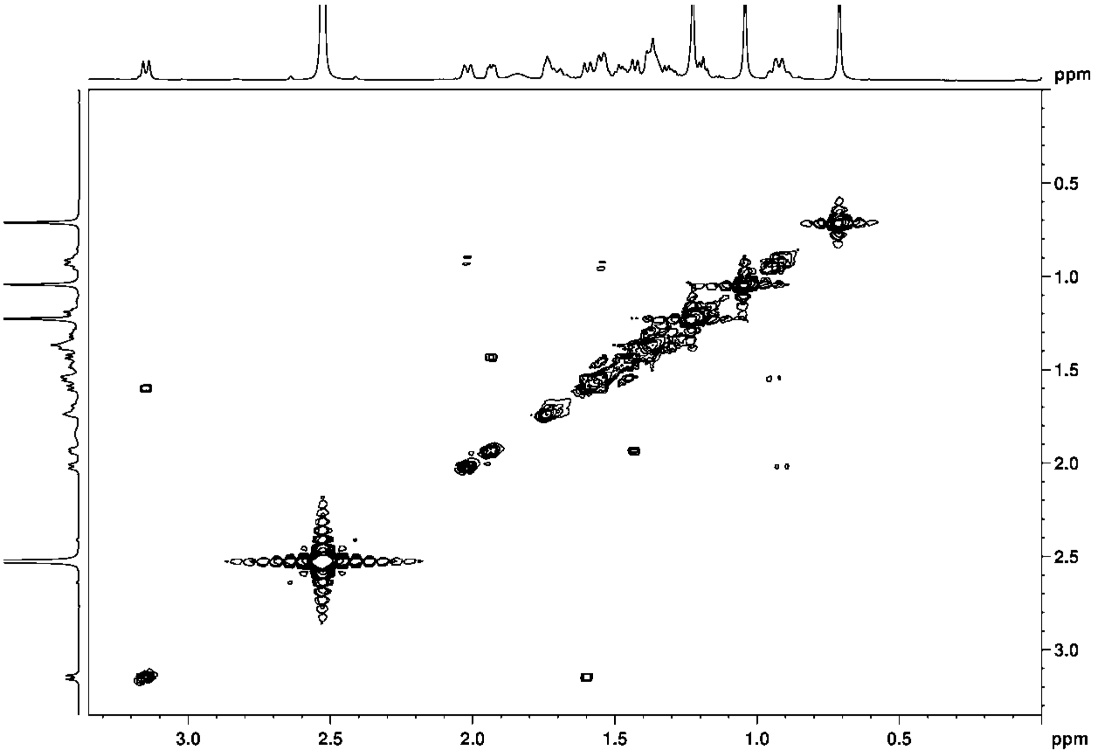


**Supplementary Fig. 17** Proton–proton Overhauser NMR spectroscopy ($^1$ H-$^1$ H NOESY spectrum, 600 MHz) of $DHGA_{12}$ in a mixture of DMSO-$d_6$/$D_2O$.

**Supplementary Fig. 18**

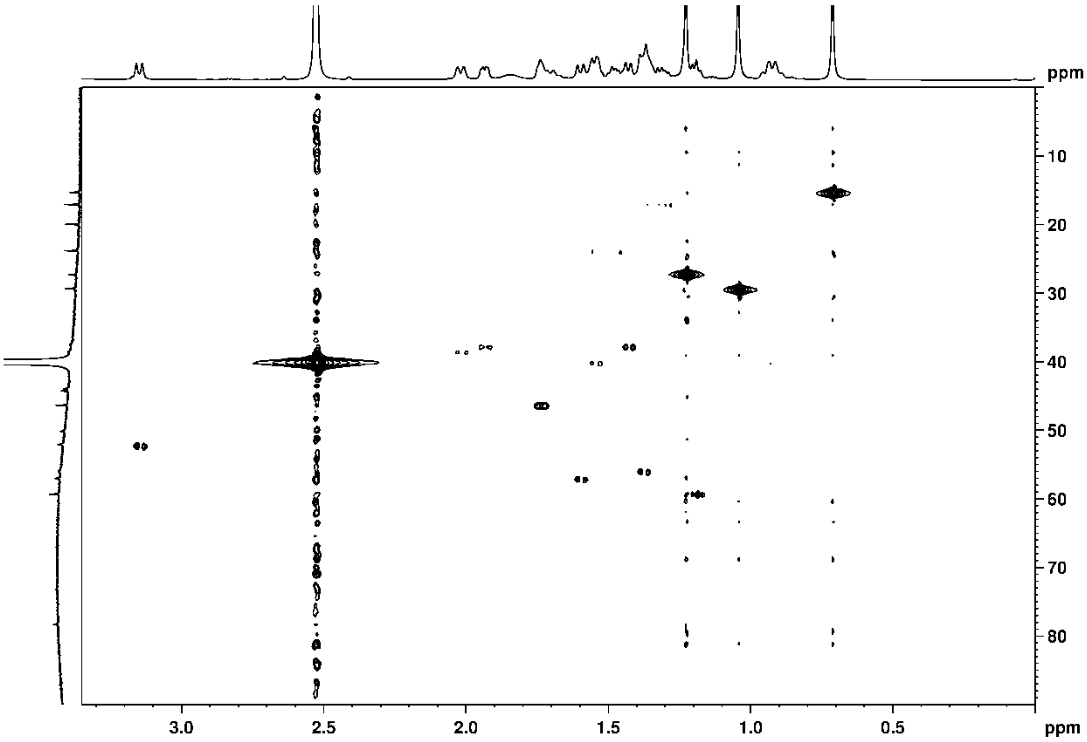


**Supplementary Fig. 18** $^{1}H$-$^{13}C$ HMQC spectrum of $DHGA_{12}$ (600 MHz) in the mixture of DMSO-$d_6$/$D_2O$.

165 **Supplementary Fig. 19**

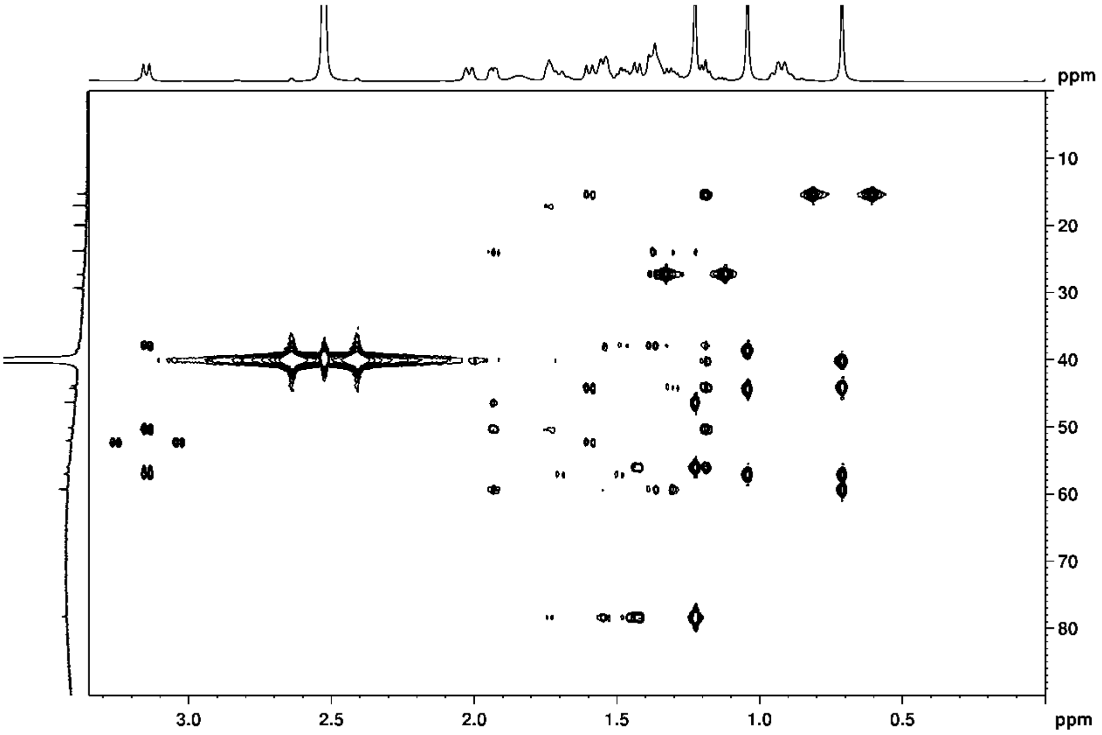


166

167

168 **Supplementary Fig. 19** $^1H$-$^{13}C$ HMBC spectrum of $DHGA_{12}$ (600 MHz) in the mixture of

169 DMSO-$d_6$/$D_2O$.

170

**Supplementary Fig. 20**

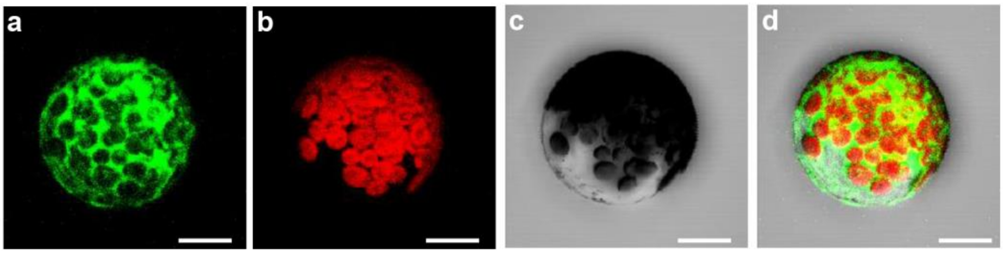


**Supplementary Fig. 20** Subcellular localization of GAS2. **a** GFP fluorescence for GAS2-GFP; **b** Chloroplast auto fluorescence; **c** Bright field image; **d** A merging image of a-c. Protoplasts were collected from 3-week-old soil-grown plants and transformed with 20 μg of plasmid DNA. Bars = 10 μm.

**Supplementary Fig. 21**

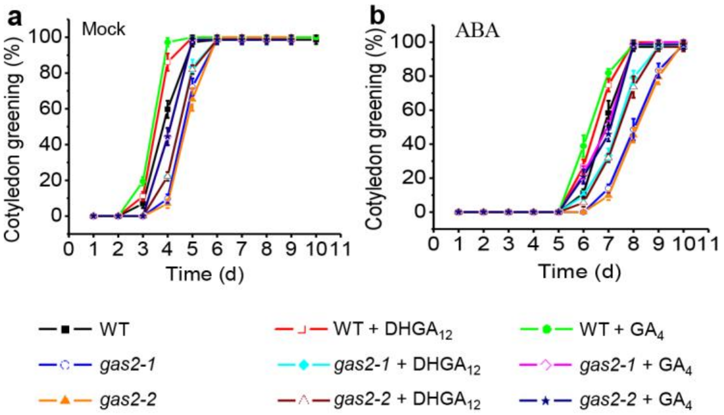


**Supplementary Fig. 21** Cotyledon greening analysis of wild type, *gas2-1* and *gas2-2* grown on MS (**a**) and MS + 0.2 μM ABA, MS + DHGA$_{12}$, MS + DHGA$_{12}$ + 0.2 μM ABA, MS + GA$_4$ and MS + GA$_4$ + 0.2 μM ABA (**b**)). Error bars represent SD (standard deviations) (n = 72). Values are the mean of three independent experiments. Source data are provided as a Source Data file.

**Supplementary Fig. 22**

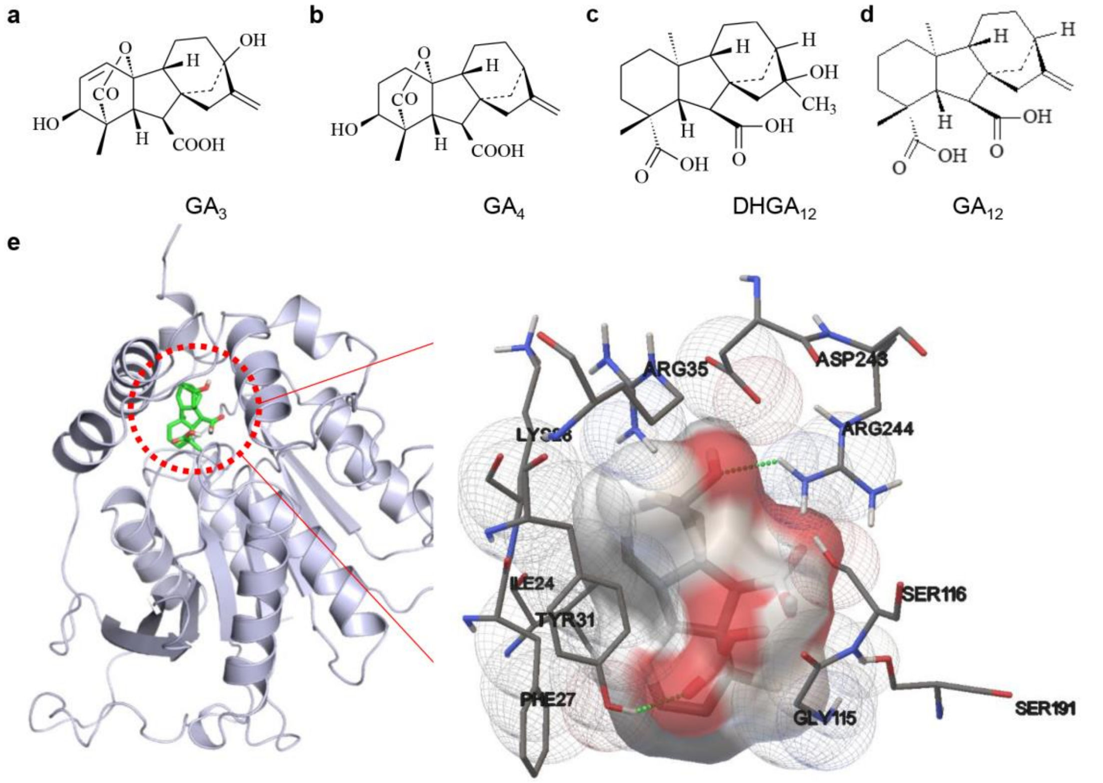


**Supplementary Fig. 22** Comparison of $GA_3$, $GA_4$, $GA_{12}$, $DHGA_{12}$ and GID1a-$DHGA_{12}$ complex in their confirmed or predicted molecular structures. **a-d** Molecular structures of previously reported GAs ($GA_3$, $GA_4$ and $GA_{12}$) and novel identified GA ($DHGA_{12}$). **e** Predicted overstructure of the GID1a-$DHGA_{12}$ complex. GID1a is shown as a cartoon and $DHGA_{12}$ as a green stick representation. A close up view of the binding region of the GID1a-$DHGA_{12}$ complex is shown at the right side. The calculated binding affinity of this interaction is -8.39 kcal/mol. The green indicates hydrogen bonds between the $DHGA_{12}$ and GID1a dashed lines.

**Supplementary Fig. 23**

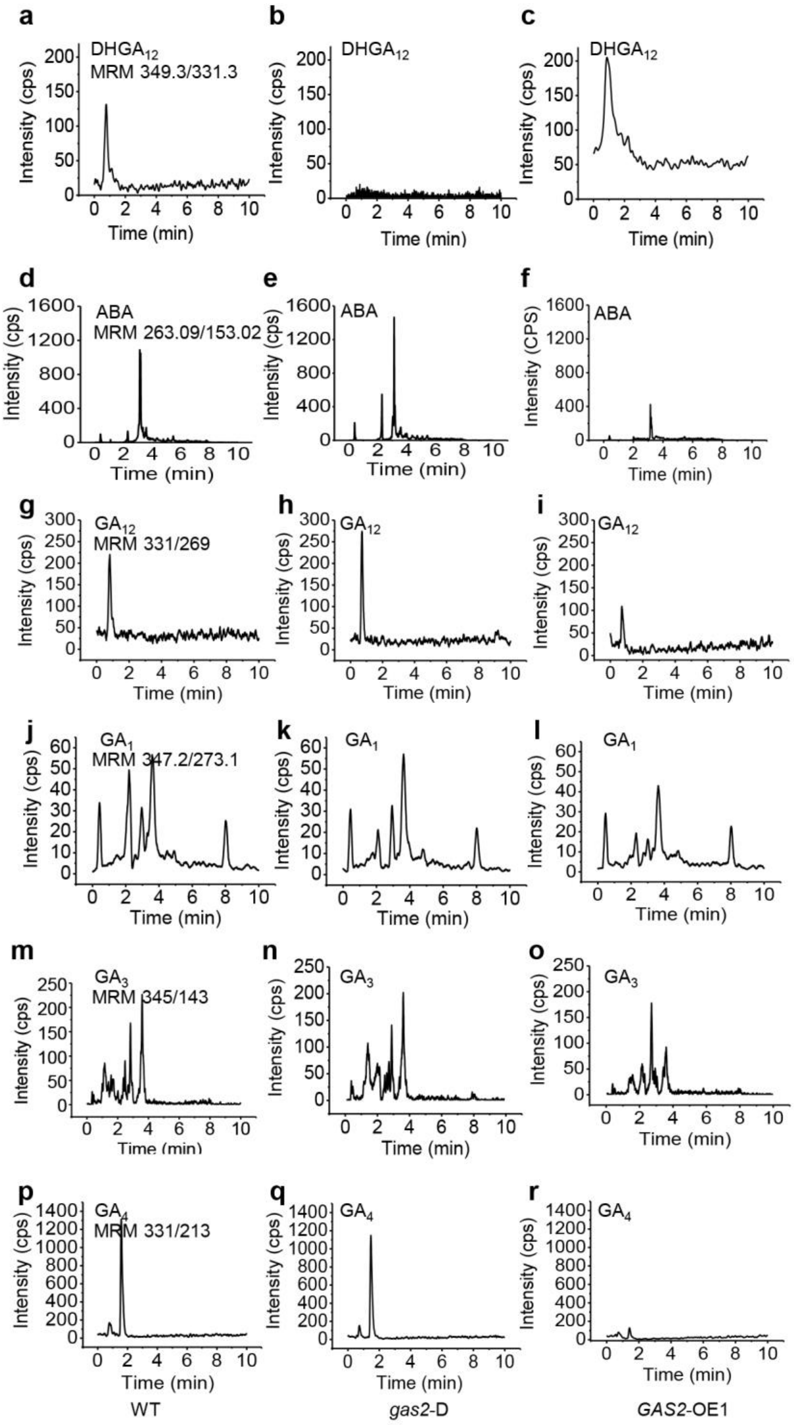


**Supplementary Fig. 23** DHGA$_{12}$, GA$_1$, GA$_3$, GA$_4$, GA$_{12}$, and ABA quantification in WT, non-induced *gas2*-D and *GAS2*-OE1. **a-r** LC-MS chromatograms of DHGA$_{12}$ (**a-c**), ABA (**d-f**), GA$_{12}$ (**g-i**), GA$_1$ (**g-l**), GA$_3$ (**m-o**), and GA$_4$ (**p-r**). The graphs from left to right were data from WT, non-induced *gas2*-D (*gas2*-D) and *GAS2*-OE1,

respectively. GAs and ABA were isolated from WT, non-induced *gas2*-D and *GAS2*-OE1. 600 mg of Arabidopsis seeds were used for each time point.

**Supplementary Fig. 24**

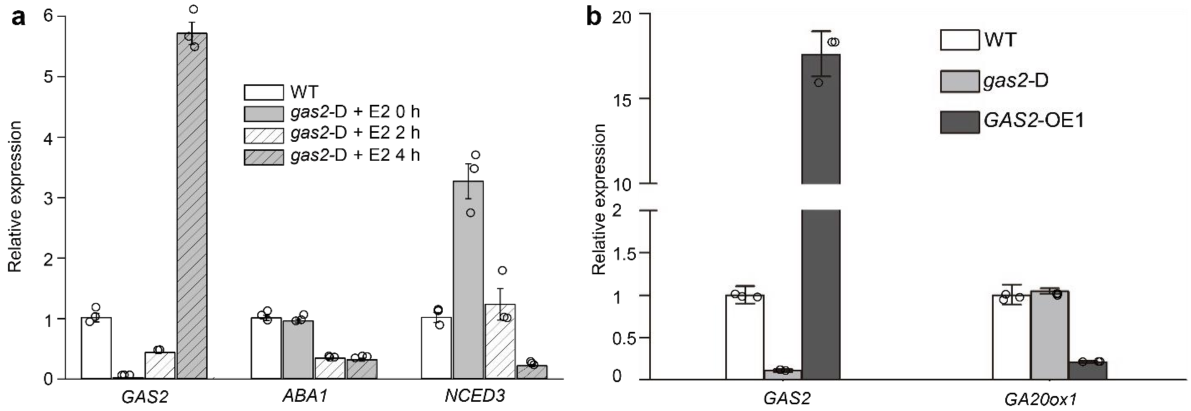


**Supplementary Fig. 24** Relative *GAS2*, *ABA1* and *NCED3* mRNA levels in WT, *gas2*-D, *gas2*-D + E2 after 2 h induction and *gas2*-D + E2 after 4 h induction (**a**). Relative *GAS*2 and *GA20ox1* mRNA levels in WT, *gas2*-D, and *GAS2*-OE1 plants (**b**). Error bars represent SD from three technical replicates. Source data are provided as a Source Data file.

**Supplementary Fig. 25**

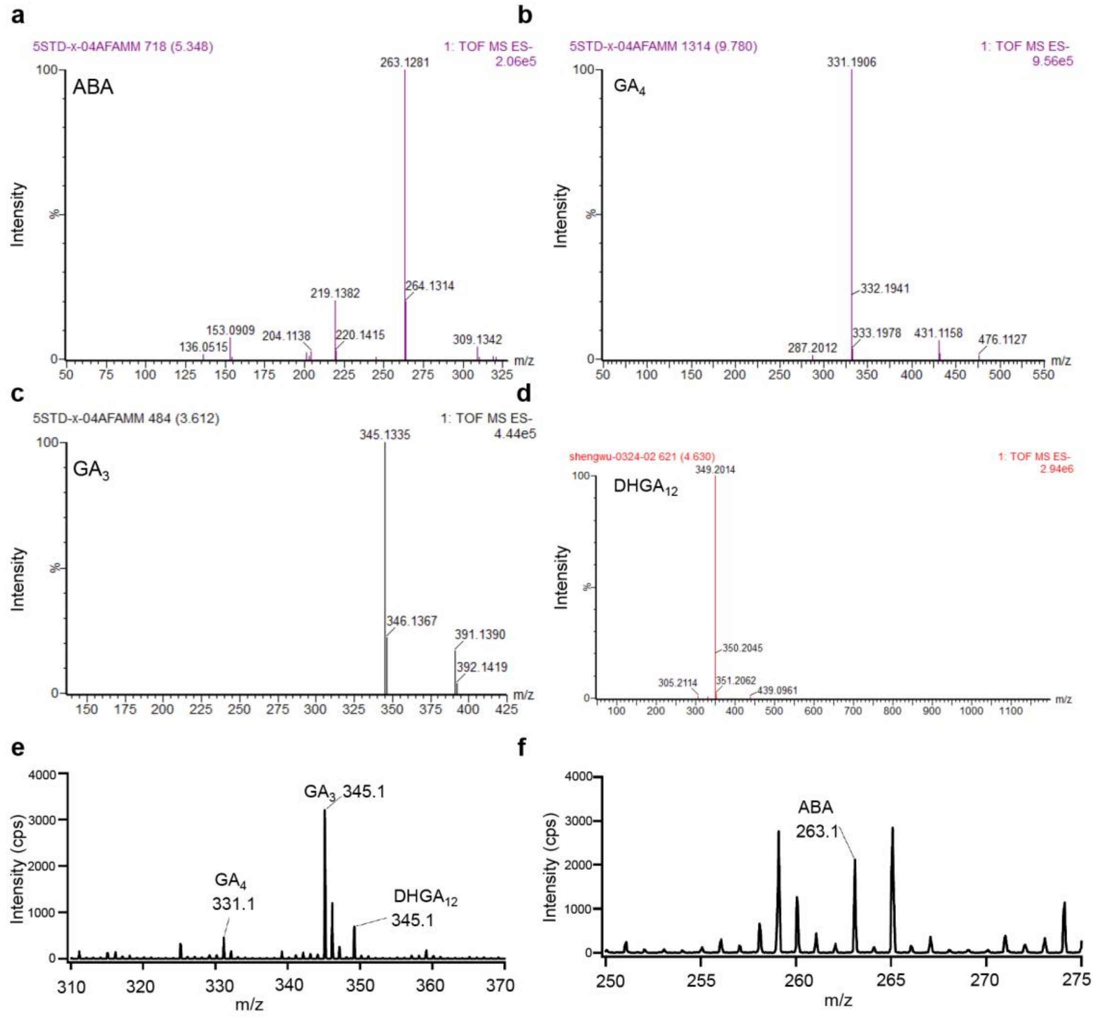


**Supplementary Fig. 25** Confirmation of the Mass-to-charge ratio (m/z) of standard and isolated compounds by Q-TOF MS and MALDI-TOF MS analysis. **a-d** Synthetic compounds **(a)** ABA standard, **(b)** $GA_4$ standard, **(c)** $GA_3$ standard and **(d)** $DHGA_{12}$. **e-f** Compounds isolated from Arabidopsis seeds $GA_3$, $GA_4$ and $DHGA_{12}$ **(e)**, and ABA **(f)**.

**Supplementary Table 1.** Metabolites identified from Arabidopsis seeds.

| Name of Metabolite | Molecular formula | Theoretical $[M-H]^-$ | Q-TOF Measured $[M-H]^-$ | MALDI-TOF Measured $[M-H]^-$ |
|---|---|---|---|---|
| ABA | C15H20O4 | 263.1283 | 263.1281 | 263.0896 |
| $GA_3$ | C19H22O6 | 345.1338 | 345.1335 | 345.1230 |
| $GA_4$ | C20H28O4 | 331.1909 | 331.1906 | 331.1398 |
| $DHGA_{12}$ | C20H30O5 | 349.2015 | 349.2014 | 349.1853 |

**Supplementary Table 2.** Primers used in this study.

| Experiment | Primer | Sequence (5’-3’) |
|---|---|---|
| Real-time PCR | qGAS2-F | CCTAACCGGCCTCATGTTCTAC |
| | qGAS2-R | TCATATTCTTGCTCACGTCTCC |
| | qUBQ10-F | CGGATCAGCAGAGGCTTATTT |
| | qUBQ10-R | GGGTGGATTCCTTCTGGATATTG |
| Genomic PCR | GAS2-F | TGCATGGCTCATATCGTTG |
| | GAS2-R | AGATGGGTCGGTCAGGTTC |
| | LBa1 (ga20ox1) | TGGTTCACGTAGTGGGCCATCG |
| | LexA3 (GAS2) | ATCATCCCCTCGACGTACTGTAC |
| | LexA4 (GAS2) | CTGGTTTTATATACAGCAGTCGACG |
| | LexA5 (GAS2) | AGTCGAGGTAAGATTAGATATGG |
| | AD1 (GAS2) | (AGCT)TCGA(G/C)T(A/T)T(G/C)G(A/T)GTT |
| | AD2 (GAS2) | NGTCGA(G/C)(A/T)GANA(A/T)GAA |
| | AD3 (GAS2) | (A/T)GTGNAG(A/T)ANCANAGA |
| | AD4 (GAS2) | AG(A/T)GNAG(A/T)ANCA(A/T)AGG |
| | 35S-GAS2-F | CCCAAGCTTATGTCGCCGTCAATGATAGCTC |
| | 35S-GAS2-R | GGGGGATCCTCATACATGGTGAATCTTGCGT |
| Subcellular localization | GAS2-pHBT-GFP-F | CCCTCTAGAATGTCGCCGTCAATGATAGCTC |
| | GAS2-pHBT-GFP-R | CCCGGTACCTACATGGTGAATCTTGCGTGAT |
| GAS2 overexpression | GAS2OE-F | CCCAAGCTTATGTCGCCGTCAATGATAGCTC |
| | GAS2OE-R | GGGGGATCCTCATACATGGTGAATCTTGCGT |
| Transient expression | 103GAS2GaF | GGGGACAAGTTTGTACAAAAAAGCAGGCTGCATGTCGCCGTCAATGATAGCTC |
| | 103GAS2GaR | GGGGACCACTTTGTACAAGAAAGCTGGGTGTACATGGTGAATCTTGCGTGATTC |
| CRISPR-cas | sgRNA | ATCACCGGAGCTATCATTGA |

| | | |
|---|---|---|
| 9 | GAS2-LP | CAACTTACCAAGTCTCCAAACATAG |
| | GAS2-RP | ATGTGCTTTAGGGTTTAGAGAACCA |
| | | GGGGACAAGTTTGTACAAAAAAGCAGGCTGC |
| *GAS2pro::G* | GAS2proGaF | GAACTGTCTTCAACCACACATTC |
| *AS2:GUS* | GAS2proGaR | GGGGACCACTTTGTACAAGAAAGCTGGGTG |
| | | TACATGGTGA ATCTTGCGTG ATTC |
| GAS2 and | GAS2-pGEX-2T-F | CTCGGATCCATGTCGCCGTCAATGATAGCT |
| GID1c | GAS2-pGEX-2T-R | GGGAAGCTTTCATACATGGTGAATCTTGCG |
| expression | GID1c–pGEX4T-F | CCGGAATTCATGGCTGGAAGTGAAGAAGTTAATC |
| in *E. coli* | GID1c –pGEX4T-R | TTTATAGCGGCCGCTCATTGGCATTCTGC |
