# Supplementary material for: Biosynthesis of DHGA12 and its roles in Arabidopsis seedling establishment
Source: Nat Commun. 2019 Apr 16;10:1768. doi: 10.1038/s41467-019-09467-5 (PMC6467921; doi:10.1038/s41467-019-09467-5)
Supplement: Supplementary file 2 — Reporting Summary [file 41467_2019_9467_MOESM2_ESM.pdf]

nature research

Corresponding author(s): Chun-Peng Song

Last updated by author(s): Feb 26, 2019

# Reporting Summary

Nature Research wishes to improve the reproducibility of the work that we publish. This form provides structure for consistency and transparency in reporting. For further information on Nature Research policies, see Authors & Referees and the Editorial Policy Checklist.

## Statistics

For all statistical analyses, confirm that the following items are present in the figure legend, table legend, main text, or Methods section.

| n/a | Confirmed | |
|---|---|---|
| ☐ | ☒ | The exact sample size (*n*) for each experimental group/condition, given as a discrete number and unit of measurement |
| ☐ | ☒ | A statement on whether measurements were taken from distinct samples or whether the same sample was measured repeatedly |
| ☐ | ☒ | The statistical test(s) used AND whether they are one- or two-sided<br>*Only common tests should be described solely by name; describe more complex techniques in the Methods section.* |
| ☐ | ☒ | A description of all covariates tested |
| ☐ | ☒ | A description of any assumptions or corrections, such as tests of normality and adjustment for multiple comparisons |
| ☐ | ☒ | A full description of the statistical parameters including central tendency (e.g. means) or other basic estimates (e.g. regression coefficient) AND variation (e.g. standard deviation) or associated estimates of uncertainty (e.g. confidence intervals) |
| ☐ | ☒ | For null hypothesis testing, the test statistic (e.g. *F*, *t*, *r*) with confidence intervals, effect sizes, degrees of freedom and *P* value noted<br>*Give P values as exact values whenever suitable.* |
| ☒ | ☐ | For Bayesian analysis, information on the choice of priors and Markov chain Monte Carlo settings |
| ☒ | ☐ | For hierarchical and complex designs, identification of the appropriate level for tests and full reporting of outcomes |
| ☒ | ☐ | Estimates of effect sizes (e.g. Cohen's *d*, Pearson's *r*), indicating how they were calculated |

*Our web collection on statistics for biologists contains articles on many of the points above.*

## Software and code

Policy information about availability of computer code

| | |
|---|---|
| Data collection | Nanotemper control software (Nanotemper Technologies); Waters MassLynx 4.1 software of Waters ACQUITY UPLC H-Class and Waters SYNAPT G2-Si HDMS; Q Exactive™ Hybrid Quadrupole-Orbitrap™ Mass Spectrometer (ThermoFisher); 9.4T Solari X MALDI-FTICR MS (Bruker). |
| Data analysis | Origin 8.0; Compass Data Analysis 5.0 (Bruker Daltonics, Billerica, MA). |

For manuscripts utilizing custom algorithms or software that are central to the research but not yet described in published literature, software must be made available to editors/reviewers. We strongly encourage code deposition in a community repository (e.g. GitHub). See the Nature Research guidelines for submitting code & software for further information.

## Data

Policy information about availability of data

All manuscripts must include a data availability statement. This statement should provide the following information, where applicable:

- Accession codes, unique identifiers, or web links for publicly available datasets
- A list of figures that have associated raw data
- A description of any restrictions on data availability

The authors declare that all relevant data supporting the findings of this study are included in the main manuscript file or Supplementary Information or are available from the corresponding author upon reasonable request. The source data of images in Figs. 1a, 1d, 5a-b, 5e, 6b and 7g as well as the source data underlying Figs. 1b-c, 1-f, 2a-g, 3e, 3j, 5c-d, 5f, 6a, 6c and 7a-f are provided in the Source Data file. For the source data of Supplementary data, the source data of the gels and images in Supplementary Figs. 1a-b and 1d-e as well as the source data underlying Supplementary Figs. 1f, 2a-d, 21a-b and 24a-b are also provided in the Source Data file.

# Field-specific reporting

Please select the one below that is the best fit for your research. If you are not sure, read the appropriate sections before making your selection.

☒ Life sciences ☐ Behavioural & social sciences ☐ Ecological, evolutionary & environmental sciences

For a reference copy of the document with all sections, see nature.com/documents/nr-reporting-summary-flat.pdf

# Life sciences study design

All studies must disclose on these points even when the disclosure is negative.

| | |
|---|---|
| Sample size | No sample-size calculation was performed. For cotyledon greening analyses, as standard, 72 seeds were used. For hypocotyl measurements, as standard, 22 seedlings were used. |
| Data exclusions | No data were excluded from analysis. |
| Replication | At least three biological replicates were performed for cotyledon greening analyses, hypocotyl measurements and transcript analyses. |
| Randomization | For cotyledon greening analyses and hypocotyl measurements, the seedlings were exposed to uniform light, seedlings were randomly picked for statistics and measurements. |
| Blinding | Not relevant to this study, because the mutants were screened by ourself that show clear phenotypes compared with the wild-type. |

# Reporting for specific materials, systems and methods

We require information from authors about some types of materials, experimental systems and methods used in many studies. Here, indicate whether each material, system or method listed is relevant to your study. If you are not sure if a list item applies to your research, read the appropriate section before selecting a response.

## Materials & experimental systems

| n/a | Involved in the study | |
|---|---|---|
| ☒ | ☐ | Antibodies |
| ☒ | ☐ | Eukaryotic cell lines |
| ☒ | ☐ | Palaeontology |
| ☒ | ☐ | Animals and other organisms |
| ☒ | ☐ | Human research participants |
| ☒ | ☐ | Clinical data |

## Methods

| n/a | Involved in the study | |
|---|---|---|
| ☒ | ☐ | ChIP-seq |
| ☒ | ☐ | Flow cytometry |
| ☒ | ☐ | MRI-based neuroimaging |
